# Screening of exogenous nutrients for pathogenic bacteria and development of highly active bactericides

**DOI:** 10.1128/msystems.01586-25

**Published:** 2026-02-12

**Authors:** Yao Ruan, Miao Zhang, Zhenyang Ge, Ting Cheng, Hao Tang, Zhi Zhang, Hailong Yu, Jie Yuan, Haoran Yin, Yiran Zhang, Shuaiyang Wang, Shengzhen Xu, Minhui Cao, Qingye Zhang

**Affiliations:** 1 National Key Laboratory of Agricultural Microbiology, National Engineering Research Center of Microbial Pesticides, Huazhong Agricultural University 47895, Wuhan, People's Republic of China; 2 Hubei Key Laboratory of Agricultural Bioinformatics, College of Informatics, Huazhong Agricultural University 47895, Wuhan, People's Republic of China; 3 College of Chemistry, Huazhong Agricultural University 47895, Wuhan, Hubei Province, People's Republic of China; 4 Hubei Hongshan Laboratory, Wuhan, People's Republic of China; Northwestern University Feinberg School of Medicine, Chicago, Illinois, USA

**Keywords:** genome-scale metabolic model, exogenous nutrients, antibiotic resistance, transmembrane transport, drug development

## Abstract

The inherent barriers posed by bacterial outer membranes, efflux pumps, and biofilm matrices significantly limit the clinical efficacy of antimicrobial agents, underscoring the urgent need for strategies to enhance drug penetration. Integrating pathogen-specific exogenous nutrients with conventional antibiotics has emerged as a promising approach, facilitating the targeted delivery and enhanced efficacy of antimicrobial compounds. In this study, we aimed to improve antimicrobial efficacy by enhancing transmembrane transport. First, we comprehensively compared various genome-scale metabolic reconstruction methods to identify the optimal approach. Subsequently, we enhanced our previous approach to identify exogenous nutrients by integrating topological screening, flux scoring, and chemical structure analysis. Key exogenous nutrients were identified for three pathogens: urea for *Acinetobacter baumannii*, acetamide for *Pseudomonas aeruginosa*, and succinic acid for *Salmonella enterica*. Growth assays confirmed that these nutrients significantly promoted bacterial proliferation. Leveraging these findings, four novel antimicrobial compounds (NC, NA, MA, and MN) were synthesized by conjugating membrane-resistant nalidixic acid or magnolol with the respective nutrients. MN enhanced the antimicrobial activity against wild-type *S. enterica* by 56.5%, while MA and NA boosted the activity against wild-type *P. aeruginosa* by 51.4% and 70.4%, respectively. Moreover, NC improved efficacy against drug-resistant *A. baumannii* by fourfold. These results demonstrate that conjugating exogenous nutrients with antibiotics can effectively enhance antimicrobial activity and help overcome membrane-associated resistance. This nutrient-conjugation strategy offers a promising avenue for developing new antimicrobial agents.

**IMPORTANCE:**

The difficulty of achieving effective drug penetration into bacterial cells is a major obstacle limiting antimicrobial efficacy and posing a significant global health challenge. This study demonstrates a novel strategy to combat resistance by “hijacking” nutrients that pathogens rely on for growth. By combining antibiotics with these nutrients, drugs can bypass membrane barriers and effectively reach their targets. The preferred exogenous nutrients of the high-priority pathogens *Acinetobacter baumannii*, *Pseudomonas aeruginosa*, and *Salmonella enterica* were identified. Combining these with the existing antibiotics markedly enhanced antimicrobial efficacy against both susceptible and resistant strains. This approach offers a practical way to revitalize existing antibiotics and design new ones, potentially slowing the spread of resistance. Importantly, it highlights how understanding bacterial metabolism can lead to smarter drug design, addressing a critical need in global health.

## INTRODUCTION

In recent decades, traditional antimicrobial agents have effectively controlled infections, but overreliance on them has spurred rapid resistance, undermining efficacy and posing risks to public health and the environment (1). For instance, *Acinetobacter baumannii* exhibits multidrug resistance and disinfectant tolerance, enabling it to persist in hospital environments and cause outbreaks (2). Similarly, the multidrug resistance of *Pseudomonas aeruginosa* complicates treatment (3). The trend of multidrug-resistant *Salmonella enterica* is also increasingly alarming, making it one of the leading bacterial causes of foodborne deaths (4). The development of novel antimicrobial agents has become an urgent global public health imperative.

One of the key reasons limiting drug efficacy is the difficulty of effectively penetrating bacterial cells. The outer membrane of gram-negative bacteria constitutes a natural chemical barrier, significantly hindering drug permeation. Additionally, the active transport mechanisms of bacterial multidrug efflux pumps rapidly expel intracellular drugs (5, 6). Moreover, the extracellular polymeric matrix produced by bacterial biofilms further establishes a robust physical barrier, markedly reducing drug penetration and diffusion, thus significantly compromising the clinical efficacy of antimicrobial (7). *P. aeruginosa* and *A. baumannii* lack non-specific channel proteins, limiting drug entry, and their double-membrane structure forms a robust barrier that enhances resistance (8, 9). *S. enterica* contributes to antibiotic resistance by regulating the expression of specific outer membrane proteins or membrane channels, thereby preventing antibiotics from entering the cells (10). Consequently, strategies aimed at overcoming bacterial membrane barriers and enhancing intracellular drug penetration are essential for improving the clinical efficacy of antimicrobial therapies.

Exogenous nutrients have recently emerged as valuable tools to address bacterial antibiotic resistance. Recent functional metabolomics studies have established a metabolic state-reprogramming paradigm in which exogenous nutrients substantially enhance the bactericidal efficacy of multiple classes of antibiotics by stimulating respiration, the proton motive force, and drug uptake in resistant pathogens (11–14). For example, exogenous alanine or glucose can restore susceptibility of kanamycin-resistant bacteria to kanamycin, and glutamine enhances antibiotic uptake and killing by multiple drug classes against multidrug-resistant gram-negative pathogens (12–14). This metabolite-based adjuvant concept has been generalized as a “metabolic state-driven” strategy and a call for next-generation agents that remove the uptake barrier to combat antibiotic resistance (11, 15). In addition to serving as antibiotic adjuvants, exogenous nutrients can also be exploited for the structural modification of existing antibiotics. For example, one study uses pathogen-preferred exogenous nutrients as synthetic precursors, covalently integrating them into bactericidal agents to construct nutrient-guided “Trojan-horse” prodrugs (16). It leverages species-specific metabolic network topology to identify exogenously required nutrients, thereby enabling species-targeted delivery and intracellular release, which mitigates membrane-associated resistance in pathogens.

Genome-scale metabolic network models (GEMs) are widely used tools that predict metabolic features based on genomic and environmental information (17). The topology of a network can directly reflect its formation and evolution (18). Topological analysis can be used to assess network robustness or metabolic phenotypes, revealing potential pathophysiological mechanisms, predicting the viability of mutant strains, and determining cellular regulation (19–22). Flux balance analysis (FBA) is a widely used method that determines optimal metabolic flux distributions by constructing a stoichiometric matrix and optimizing an objective function (23). The metabolic flux distribution obtained by solving traditional FBA is often not unique. This issue can be resolved by incorporating a secondary optimization criterion, such as total flux minimization (pFBA), flux sampling, or flux variability analysis (FVA) (24). Flux-based analysis can predict whether an organism can absorb nutrients. Automated modeling tools and curation reconstruction tools have been developed, such as KBase, CarveMe, and DEMETER (25). The human gut microbiome GEMs reconstructed using the KBase and DEMETER pipeline are of high quality and exhibit accurate predictions (26, 27). The automated GEM reconstruction tool CarveMe is also widely used in microbial GEM reconstructions due to its high consistency with manually curated networks (28, 29).

This study aims to design novel bactericides based on exogenous nutrients to overcome transmembrane transport challenges. First, we select an appropriate microbial GEM reconstruction method based on experimental data, followed by the optimization of the screening method, which integrates topological screening, flux scoring, and chemical structure analysis, further improving the prediction accuracy. We selected three pathogens with severe transmembrane difficulty issues as application examples: *P. aeruginosa*, *A. baumannii*, and *S. enterica*. By utilizing their exogenous nutrients, we modified existing antimicrobial agents. This modification significantly enhanced drug entry into the bacteria, resulting in improved bactericidal effects and effectively addressing resistance problems caused by transmembrane transport challenges.

## RESULTS

### Screening the best GEM reconstruction methods for pathogenic bacteria

To accurately identify exogenous nutrients in microbes, it is necessary to select a GEM reconstruction method with high modeling quality. Given the automation strengths of KBase and CarveMe and DEMETER’s improvements in GEM quality, these pipelines are strong candidates. Consequently, the following four approaches were selected to compare and identify the most suitable method for reconstructing microbial GEMs to detect exogenous nutrients: KBase, CarveMe, DEMETER Refined KBase, and DEMETER Refined CarveMe. The accuracy of these modeling methods was then compared based on three aspects: stoichiometric and flux consistency, the accuracy of predictions for metabolite secretion and uptake, and the accuracy of predictions for auxotrophs. Although experimental data on exogenous nutrients remain limited, the requirement for exogenous nutrients (auxotrophy) has been experimentally validated in seven microbial species (30). The GEMs of these seven species were reconstructed for method comparison: *Clostridium* sp. L2-50, *Coprococcus catus* GD/7, *Eubacterium hallii* DSM 3353, *Eubacterium rectale* M104/1, *Faecalibacterium prausnitzii* SL3/3, *Roseburia intestinalis* L1-82, and *Subdoligranulum variabile* DSM 15176.

**Fig 1:**
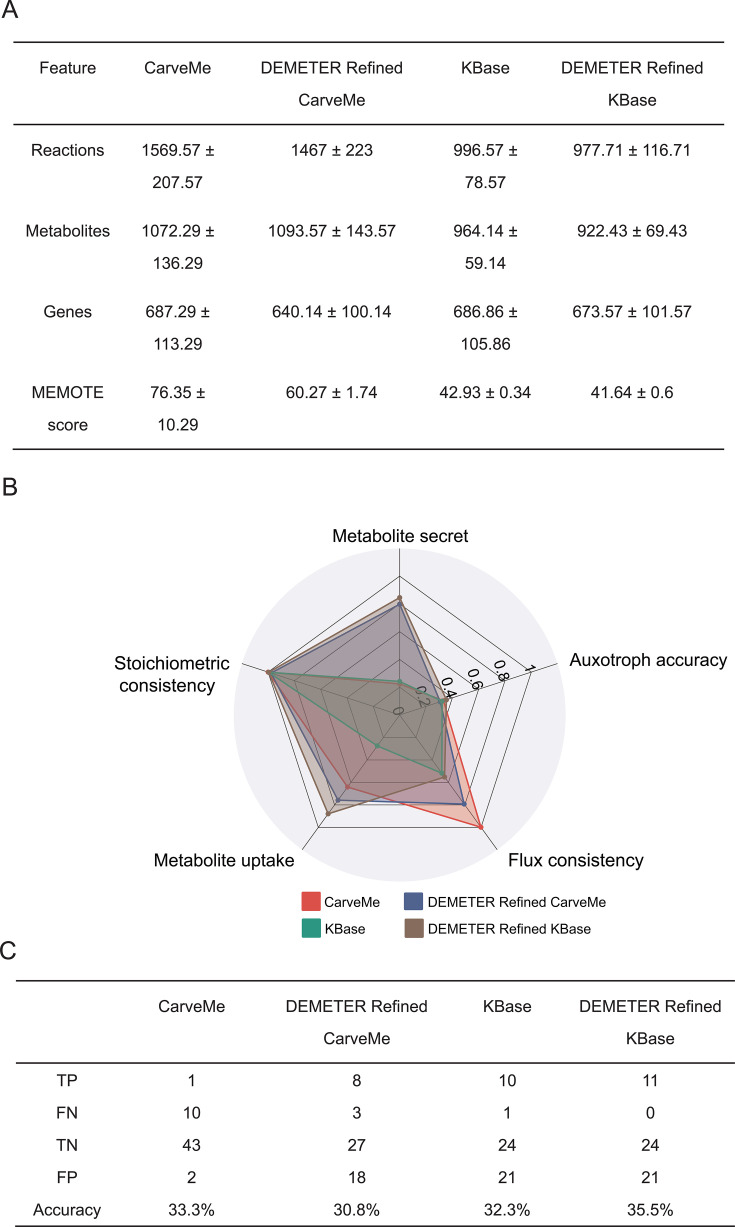
Comparison of GEMs reconstructed using four modeling methods. (**A**) Average number of metabolites, reactions, genes, and average MEMOTE scores in GEMs reconstructed for seven microbes using four different modeling methods. (**B**) Comparison of accuracy for GEMs reconstructed using four modeling methods, with averages taken across various metrics for microbial GEMs. (**C**) Comparison of the prediction accuracy for nutrient deficiencies among GEMs constructed using four modeling methods. TP, true positive; FN, false negative; TN, true negative; FP, false positive.

The GEMs reconstructed using the four methods exhibited varying numbers of metabolic reactions and genes, as shown in Fig. 1A. Metabolic networks optimized with DEMETER generally exhibited lower numbers of reactions, metabolites, and genes compared to those that were not optimized. Due to CarveMe-reconstructed GEMs having more annotations for reactions, metabolites, and genes, they achieve higher MEMOTE scores compared to other methods (Fig. 1A).

To validate the accuracy of predictions for secretion and uptake, experimental data on microbial metabolite secretion and uptake from the NJC19 data set were used (31). Exchange reactions served as the objective function for FBA simulations, allowing us to determine secretion (flux > 0) and uptake (flux < 0). As shown in Fig. 1B, all four modeling methods demonstrated excellent stoichiometric consistency, exceeding 99%. The DEMETER Refined KBase method demonstrated superior performance, achieving the highest average accuracies in predicting metabolite secretion and uptake. Additionally, it recorded the highest overall prediction accuracy for predicting auxotroph types. Radar charts for GEM quality evaluation indicators for each microorganism are presented in Fig. S1 to S6. As shown in Fig. 1C, the DEMETER Refined KBase method successfully predicted all validated nutrient deficiencies, while other methods missed some auxotroph types. The GEM reconstruction DEMETER Refined KBase method demonstrated the best overall performance among all tested methods, showing the highest consistency with experimental results. Consequently, this approach was selected as the preferred method for reconstructing GEMs to identify the exogenous nutritional requirements of microbes.

### Reconstruction and validation of GEMs for target pathogens

**Fig 2:**
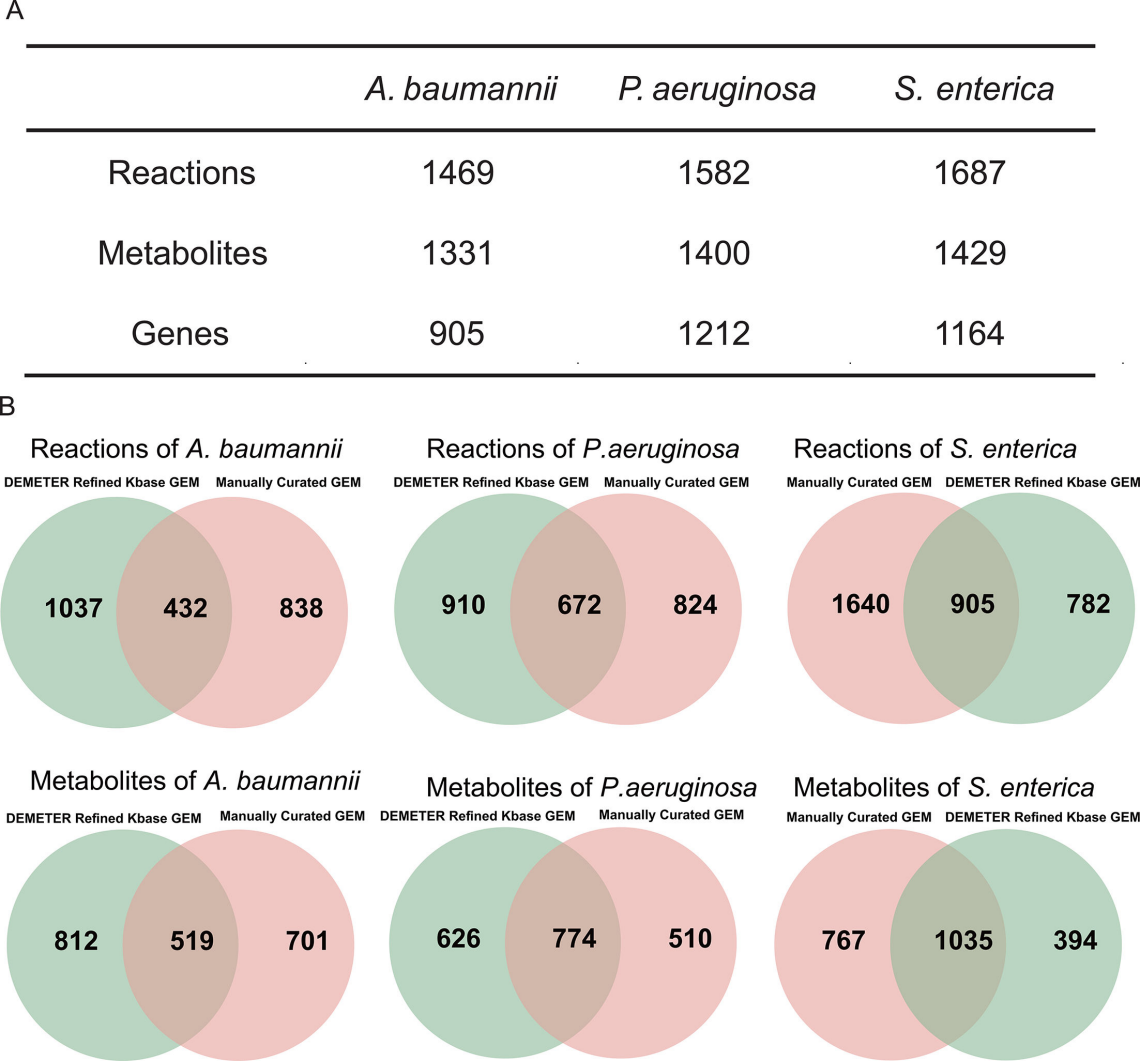
Overview of the GEMs for the three pathogens. (**A**) Number of metabolites, reactions, and genes in the GEMs of three pathogens reconstructed using the DEMETER Refined KBase method. (**B**) Venn diagram of metabolites and reactions between DEMETER Refined KBase GEMs and manually curated GEMs across three pathogenic species.

The DEMETER Refined KBase method was employed to reconstruct GEMs for three highly resistant pathogenic bacteria: *P. aeruginosa*, *A. baumannii*, and *S. enterica*. The GEM of *A. baumannii* exhibits the lowest number of reactions, metabolites, and genes, likely due to its comparatively smaller genome size (Fig. 2A). These GEMs were compared with manually reconstructed GEMs for *P. aeruginosa* iPae1146 (32), *A. baumannii* iATCC19606 (33), and *S. enterica* STM_v1_0 (34). The GEMs reconstructed using the DEMETER Refined KBase method share metabolites and reactions with previous models while also possessing many unique metabolites and reactions (Fig. 2B).

The predictive accuracy of nutrient utilization for three pathogenic bacteria was further validated. In the models, exchange reactions were designated as objective functions for FBA, with a negative flux indicating metabolite utilization. Experimental data on carbon source utilization by these pathogens were gathered to corroborate the predictions. Validation of the GEM predictions for carbon source utilization was supported by data from Zhu et al*.* (33, 35) and AbuOun et al*.* (36). As shown in Table 1, the GEMs reconstructed by automation demonstrated high accuracy in predicting carbon sources. Although the GEM for *P. aeruginosa* showed slightly lower accuracy compared to the manually curated GEM, the other two automated GEMs exhibited higher accuracy in carbon source prediction than the manually curated one. This indicates that the GEMs reconstructed using the selected methods in this study possess high predictive accuracy and can be effectively used to predict exogenous nutrients for pathogenic bacteria. Furthermore, the automated approaches can be applied to the reconstruction of GEMs for a broader range of pathogens.

**TABLE 1:** Accuracy of carbon source utilization prediction by DEMETER Refined KBase and manually curated GEM

Bacterium	DEMETER Refined KBase	Manually curated GEM
*P. aeruginosa*	0.94	0.95
*A. baumannii*	0.92	0.86
*S. enterica*	0.93	0.9

### Identification and verification of exogenous nutrients

**Fig 3:**
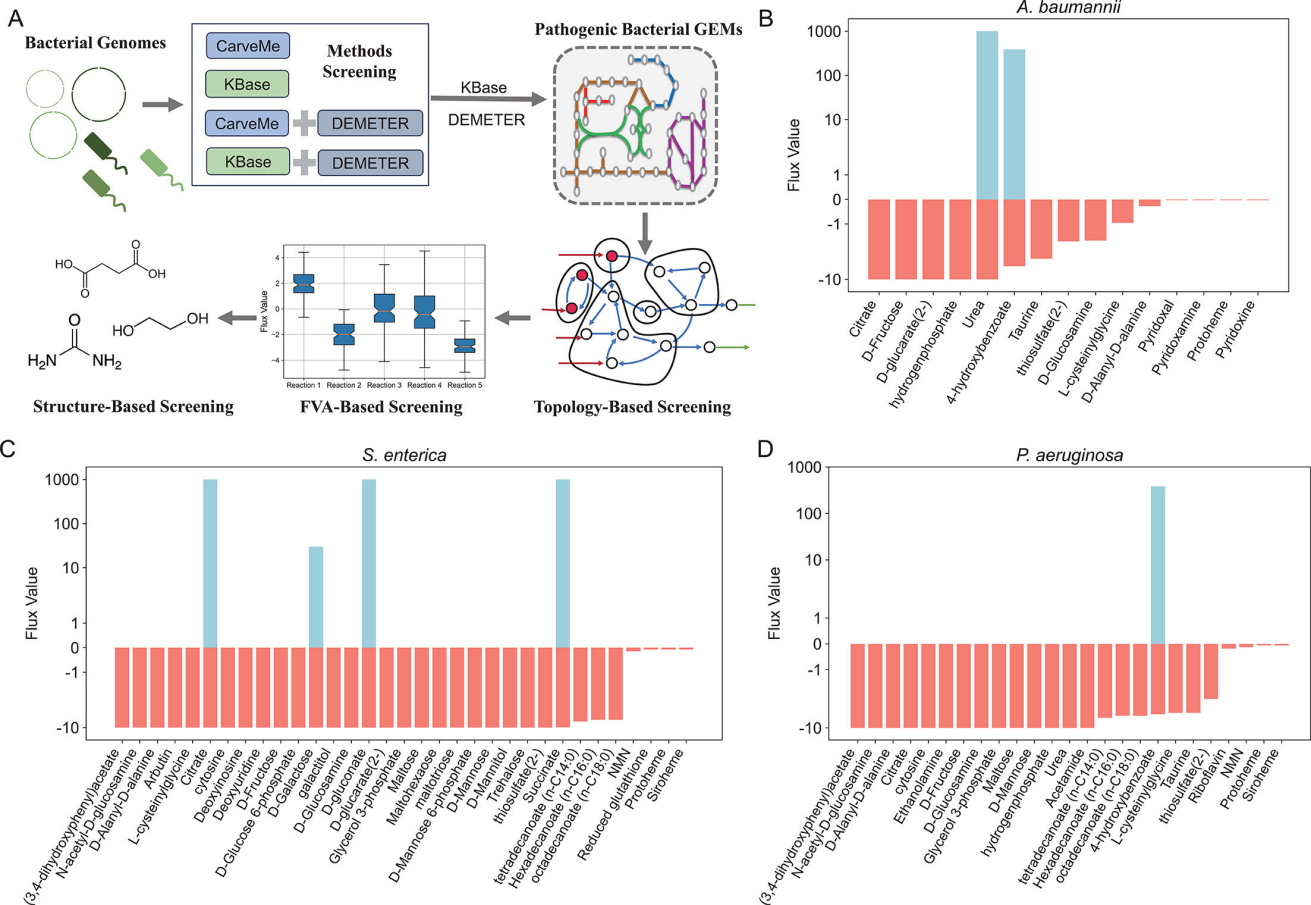
Screening exogenous nutrients. (**A**) Exogenous nutrient screening workflow, including bacterial genome acquisition, selection of a GEM reconstruction approach, reconstruction of pathogen-specific GEMs, and topology-based, FVA-based, and structure-based screening of exogenous nutrients. (**B–D**) FVA of exchange reactions corresponding to exogenous nutrients for *A. baumannii* (**B**), *S. enterica* (**C**), and *P. aeruginosa* (**D**). Bars show feasible flux ranges; negative minima indicate potential uptake capacity.

Exogenous nutrients are defined as essential nutrients that a pathogen can obtain only from the external environment. Previous topological methods identified exogenous nutrients as the potential nutritional requirements of microorganisms in various environments, but their importance for growth remains uncertain (22). This study enhances the previous approach by not only considering the topological structure but also using metabolic models to calculate the uptake rates of various exogenous nutrients by pathogenic bacteria and to evaluate their importance. Absorption rates were defined by the flux of the corresponding exchange reactions, and FVA determined these flux ranges to address FBA’s multiple solution issue; a minimum flux below zero indicates nutrient uptake (37). Following validation of the GEMs for carbon source utilization in three pathogens, the exogenous nutrients for each pathogen were identified as depicted in Fig. 3A. The pathogen GEMs were converted into directed acyclic graphs and subjected to strongly connected component (SCC) decomposition to pinpoint nutrients requiring external uptake (38). FVA was then applied to the corresponding exchange reactions, filtering out those with minimum fluxes below zero (see Supplemental Data). Ultimately, metabolites containing amino, carboxyl, and hydroxyl groups were selected as candidates for modification (see Materials and Methods).

Through topology-based screening, 84 exogenous nutrients were identified for *A. baumannii*; 56 had minimum flux < 0, and structure-based screening further prioritized 15 candidates (Fig. 3B). For *S. enterica*, 86 exogenous nutrients were identified, 72 with minimum flux < 0, yielding 33 candidates (Fig. 3C). In *P. aeruginosa*, 80 exogenous nutrients were identified, with 60 showing minimum flux < 0, resulting in 25 candidates (Fig. 3D). Detailed data are provided in the Supplemental Data.

**Fig 4:**
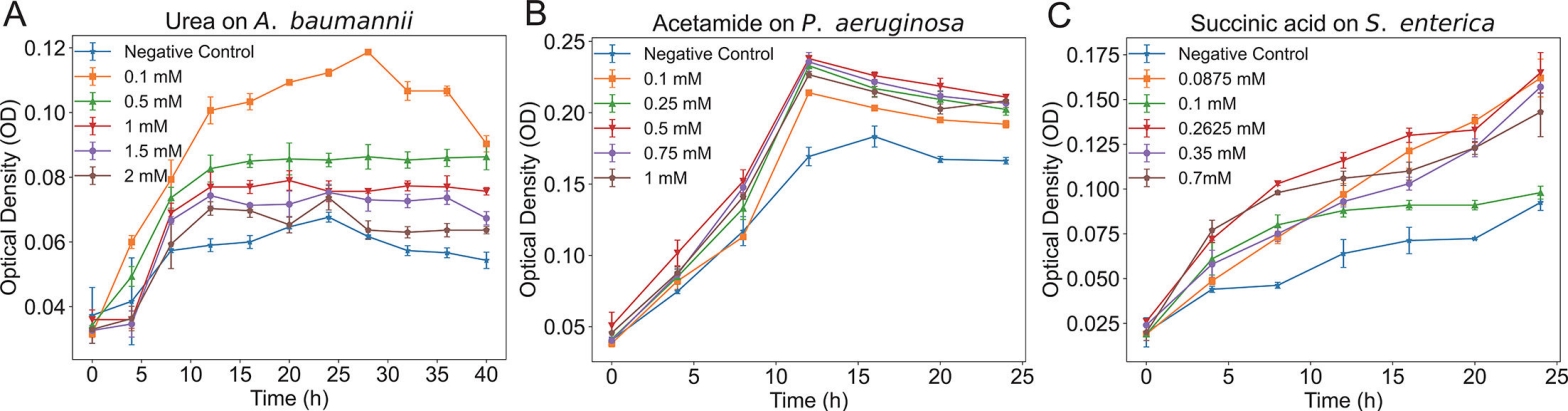
Effect of different exogenous nutrient concentrations on the growth of three species: urea on *A. baumannii* (**A**), acetamide on *P. aeruginosa* (**B**), and succinic acid on *S. enterica* (**C**).

To validate the accuracy of the results, one exogenous nutrient was selected from the identified candidates based on its high ranking in absolute flux value and supporting literature indicating its potential as a carbon or nitrogen source for pathogenic bacteria (39–41). The selected nutrient was experimentally validated through growth curve analysis. These exogenous nutrients include urea for *A. baumannii*, acetamide for *P. aeruginosa*, and succinic acid for *S. enterica*. The growth curve results indicated that the most significant promotional effect on *A. baumannii* was observed at a urea concentration of 0.1 mM/L; and then, this promotional effect gradually diminished with increasing concentrations (Fig. 4A). For acetamide, the maximum growth promotion on *P. aeruginosa* was achieved at 0.5 mM/L (Fig. 4B), while for succinic acid, the most notable effect on *S. enterica* was at 0.2625 mM/L (Fig. 4C). These findings demonstrate that the three pathogenic bacteria can effectively absorb their corresponding exogenous nutrients, which significantly promote their growth and indicate the accuracy and effectiveness of the exogenous metabolite identification method used in this study.

### Design and synthesis of target compounds

Growth curve analysis has confirmed the requirement of exogenous nutrients for the growth of three pathogenic bacteria. The potential for enhancing antimicrobial activity and addressing resistance issues by combining these nutrients with existing antimicrobial drugs will be further explored. Specifically, nalidixic acid (N) and magnolol (M) were selected for modification due to N’s high resistance and M’s poor transmembrane transport (42, 43). By linking them with exogenous nutrients via ester or amide bonds, four target compounds were successfully synthesized: NC, NA, MA, and MN, as shown in Fig. 4. These target compounds leverage special uptake pathways of exogenous nutrients to enhance their transmembrane capabilities (44).

**Fig 5:**
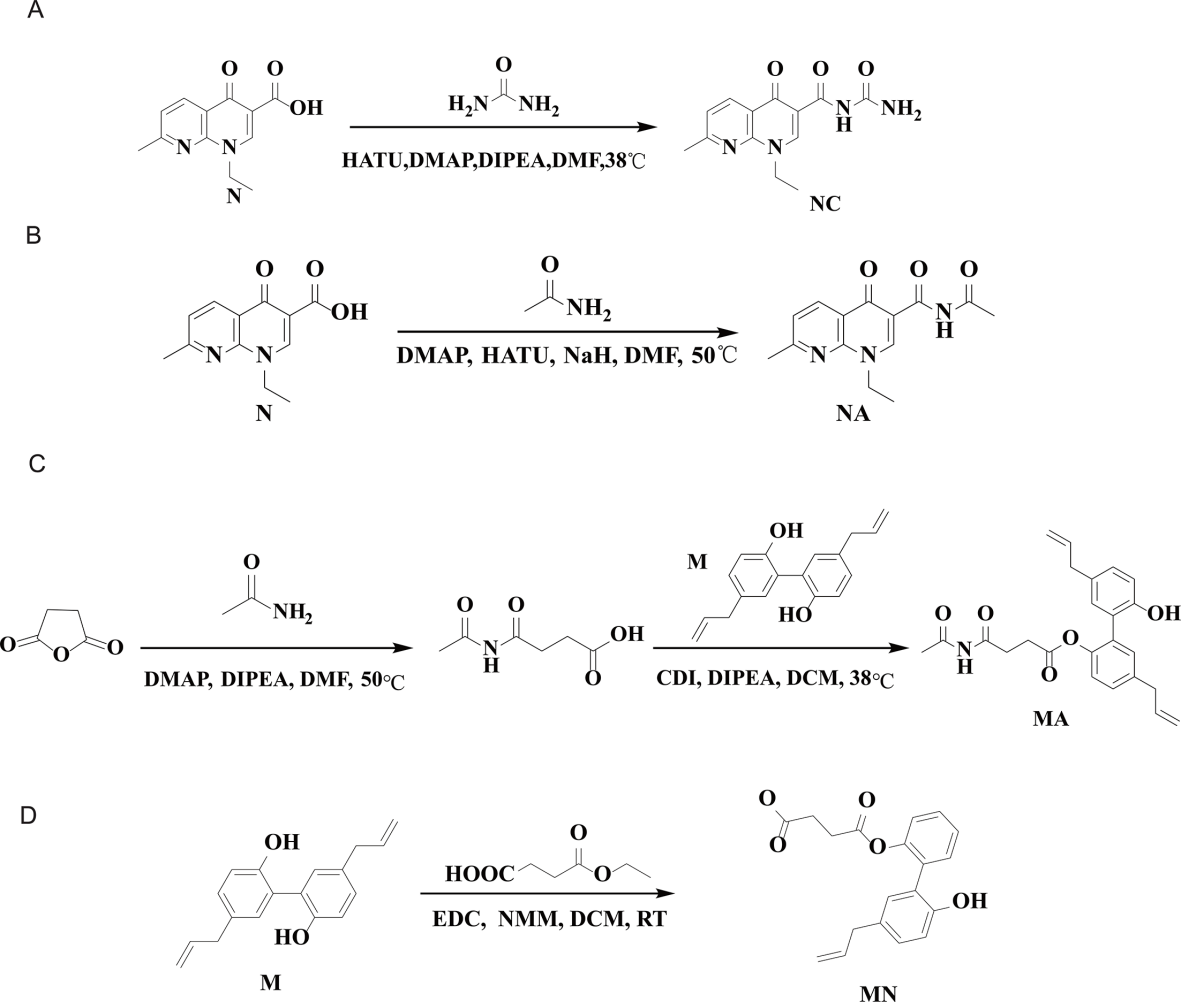
Synthetic routes for the target compounds: (**A**) NC, (**B**) NA, (**C**) MA, and (**D**) MN.

The synthesis of compound NC began with the acyl activation of N using DMAP and HATU in DMF at 40°C, followed by the addition of urea and DIPEA. Urea acted as an amino donor, forming an amide bond with N at 50°C. The reaction progress was monitored via thin-layer chromatography, and the final pale-yellow solid product, NC, was obtained through column chromatography (Fig. 5A).

The synthesis of compound NA involved mixing N, DMAP, and HATU in dichloromethane, followed by the addition of acetamide dissolved in DMF. After heating and washing, the reaction mixture was rapidly purified by column chromatography, and the target compound was dried using a rotary evaporator at 45°C (Fig. 5B).

The synthesis of the target compound MA involved two main steps. First, succinic anhydride was activated with DMAP and acetamide in the presence of triethylamine, followed by purification and drying to yield intermediate I. In the second step, intermediate I was reacted with N,N′-carbonyldiimidazole and compound M, resulting in the formation of the target compound MA as the final product (Fig. 5C).

The target compound MN was synthesized by dissolving monoethyl succinate, EDC, and NMM in DCM, followed by the addition of M. The reaction was quenched with ice-cold brine, then subjected to extraction and chromatography (Fig. 5D). The detailed synthetic procedures for the four compounds are provided in the Supplemental Methods. Complete structural characterization of the four compounds, including mass spectrometry, ¹H NMR, ¹³C NMR spectra, and infrared spectra, is provided in Fig. S7 to S26. HPLC purities (area %) were NC 97.2, NA 98.7, MA 98.8, and MN 98.5 (Fig. S23 to S26).

### Bactericidal activity test

**Fig 6:**
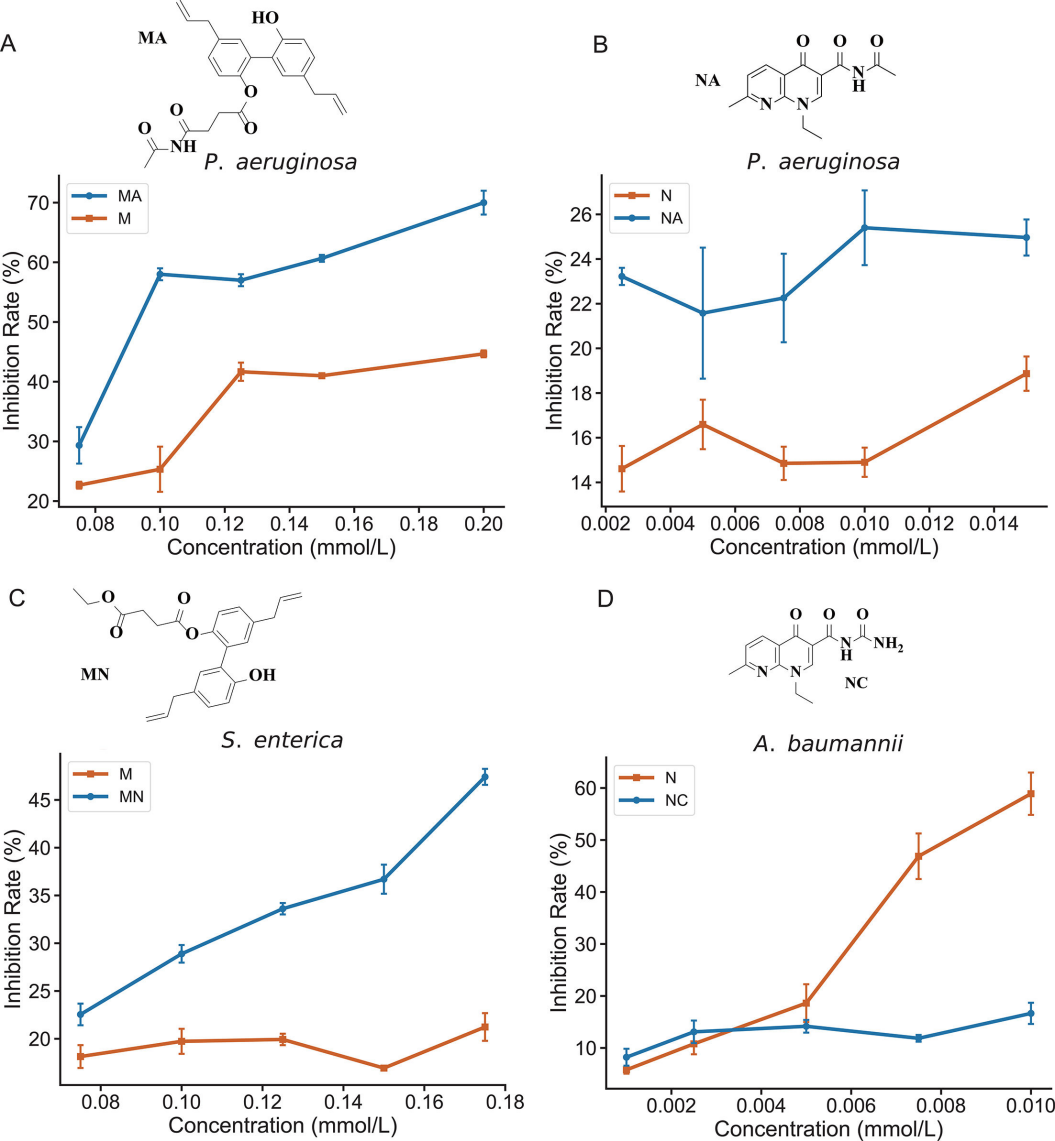
Bactericidal activity testing of target compounds. (**A**) MA and M against *P. aeruginosa*. (**B**) NA and N against *P. aeruginosa*. (**C**) M and MN against *S. enterica*. (**D**) N and NC against *A. baumannii*. Orange lines indicate inhibition by the parent compounds, whereas blue lines indicate inhibition by the modified compounds.

To verify the efficacy of the target compounds synthesized in this study, their bactericidal activity against the corresponding pathogens was tested at different concentrations. In wild-type strains, the parent drug displays a low minimum inhibitory concentration (MIC) due to immediate activity, whereas exogenous nutrient–antimicrobial conjugates require time-consuming intracellular hydrolysis and therefore exhibit higher MICs. Accordingly, potency was quantified using OD_600_-based EC_50_ values derived from fitted dose–response curves to enable a fair comparison. EC_50_ values were reported only for compounds exhibiting a monotonic, quantitative dose–response that allowed a stable fit. When no measurable dose dependence was observed, EC_50_ was not determined, and inhibition was summarized by concentration. In wild-type *P. aeruginosa*, MA showed increased potency relative to M, reflected by a 51.4% reduction in EC_50_ (M: 0.2186 mM; MA: 0.1063 mM; Fig. 6A). For the same strain, NA achieved 70.4% inhibition at 0.01 mM (Fig. 6B). In wild-type *S. enterica*, MN exhibited increased potency, with a 56.5% reduction in EC_50_ compared with M (M: 0.5432 mM; MN: 0.2363 mM; Fig. 6C).

However, in tests with the *A. baumannii* wild-type strain, compound NC did not show an improved inhibitory effect (Fig. 6D). This outcome is likely because NC acts as a prodrug that must enter the cell and undergo enzymatic hydrolysis to release the parent drug before exerting potent antibacterial effects. Hydrolysis takes time: by the standard OD_600_ endpoint, insufficient parent drug has been released; by the time hydrolysis is complete, the pathogen has already multiplied substantially, and OD_600_ remains elevated due to light scattering from cells and cellular debris, yielding only modest apparent potency.

By contrast, resistant *A. baumannii* exhibits membrane-associated resistance to the unmodified drug, so exposure to the unmodified drug alone results in prolonged bacterial survival. The conjugate (NC) can enter cells and, after prolonged incubation, release the active agent via intracellular hydrolysis, making MIC readouts based on extended growth/no-growth criteria rather than turbidity alone more informative for detecting the conjugate’s advantage. To test this hypothesis, N and NC were applied separately to drug-resistant *A. baumannii* strains.

To assess the efficacy of compounds N and NC against resistant strains of *A. baumannii*, MIC experiments were conducted using the standard strain *A. baumannii* ATCC 11038 and a quinolone-resistant strain, *A. baumannii* HZ2021-0271. The results revealed that the MIC for compound N was 0.3125 mM, while the MIC for compound NC was only 0.078125 mM, which is one-fourth that of N. These findings demonstrate a significant inhibitory effect of NC on the resistant *A. baumannii* strain (see Table 2).

**TABLE 2:** MIC experiments of compound N and NC^*a*^

Compound	MIC (mM）	
	*A. baumannii* (ATCC11038)	*A. baumannii* (HZ2021-0271)
N	0.04	0.3125
NC	>0.64	0.078125

^*a*^ SD = 0 due to identical triplicate MICs from a discrete twofold dilution grid.

## DISCUSSION

In previous work, we successfully identified the exogenous nutrients required by pathogens using metabolic network analysis. Building on this, the present study further optimized the screening process for exogenous nutrients. By comparing the predictive accuracy of various modeling methods, we found that the DEMETER Refined KBase method demonstrated the highest accuracy in identifying exogenous metabolites, with minimal deviation from experimental data. Following the selection of an optimal microbial GEM reconstruction method, we introduced a novel approach that integrates topological filtering, flux scoring, and structural screening. This combination significantly enhances the identification process of exogenous nutrients, ultimately improving the accuracy of predictions.

Unlike the adjuvant paradigm, in which metabolites are co-administered to reprogram pathogens from resistant to susceptible metabolic states, the present approach employs exogenous nutrients as synthetic precursors, covalently appended to bactericidal agents to generate nutrient-guided conjugates that are actively transported and released intracellularly. Notably, these exogenous nutrient-guided conjugates can partially overcome membrane-associated barriers and increase intracellular exposure in resistant strains, while also retaining strong activity against wild-type strains, indicating cross-phenotype applicability. Overall, metabolically driven adjuvant strategies and nutrient-guided prodrug strategies are complementary: the former provides phenotype-oriented sensitization cues, whereas the latter enables species-precise delivery and intracellular release. Combining the two, for example by incorporating metabolites identified by metabolic state reprogramming, may be particularly powerful. When such metabolites are also essential or strongly growth-supporting for the pathogen, conjugation is expected to leverage both active, growth-coupled uptake and resistance reversion, potentially yielding superior efficacy.

The outer membrane permeability of bacteria plays a pivotal role in the effectiveness of bactericidal agents. Our findings suggest that modifying bactericides with exogenous nutrients overcomes resistance associated with membrane permeability and transmembrane transport barriers by exploiting the natural nutrient uptake of pathogens, thereby enhancing drug delivery. Compared to *Enterobacteriaceae*, *P. aeruginosa* and *A. baumannii* have lower permeability to hydrophilic molecules due to the absence of non-specific large-pore proteins such as *OmpF* and *OmpC* (42). Their bilayer membranes further increase resistance, posing challenges in developing effective antimicrobials (9). Additionally, *S. enterica*, another pathogen with significant health concerns and multidrug resistance (45), was included in this study to verify the general applicability of our methods. The screening process designed in this study successfully identified suitable exogenous nutrients for each bacterium: acetamide is known to be a carbon source for *P. aeruginosa* (39), urea has been proven to support the growth of *Bacillus cereus* strain DRY135 (40), and succinate is also a carbon source that *S. enterica* can utilize (41). Growth curve experiments confirmed that these exogenous nutrients significantly promote the growth of these pathogens, further validating the effectiveness and applicability of our screening method. In future work, it will be important to expand the evaluation to a broader range of clinically relevant pathogens and resistance phenotypes to more comprehensively define the spectrum, robustness, and potential therapeutic utility of these compounds.

We addressed the challenges of membrane resistance and transmembrane transport difficulties faced by the compounds nalidixic acid and magnolol by adopting a chemical modification strategy. We synthesized four target compounds by linking them with specific exogenous nutrients of three pathogenic bacteria through ester or amide bonds. After evaluating the *in vitro* antibacterial activity, we observed that the modified compounds MA, MN, and NA demonstrated significantly enhanced effects in inhibiting pathogens compared to the original compounds. Notably, although compound NC was less effective against wild-type *A. baumannii* than the original compound, it showed a significant increase in inhibitory efficiency against multidrug-resistant strains. These results not only demonstrate the effectiveness of using exogenous nutrients for compound modification but also reveal the potential application of this method in addressing pathogen resistance issues. This work, therefore, provides a novel approach for the rational design and development of antimicrobial drugs. In future studies, it will be important to elucidate the underlying mechanisms in greater detail, for example, by tracking the uptake, intracellular distribution, and release of nutrient–antibiotic conjugates. Such investigations will help distinguish the contribution of the conjugates themselves from any metabolite–antibiotic combination effects and further refine this design framework.

This study demonstrates that employing exogenous nutrients for compound modification not only overcomes fungicide transmembrane transport challenges but also offers a versatile strategy for designing pathogen-specific bactericidal agents that expedite antimicrobial optimization, enhance efficacy, and mitigate resistance.

## MATERIALS AND METHODS

### Genome resources

Genome assemblies were retrieved from the NCBI RefSeq database as follows: *Clostridium* sp. L2-50 (GCF_000154245.1), *Coprococcus catus* GD/7 (GCF_000210555.1), *Eubacterium hallii* DSM 3353 (GCF_000173975.1), *Eubacterium rectale* M104/1 (GCF_000209955.1), *Faecalibacterium prausnitzii* SL3/3 (GCF_000209855.1), *Roseburia intestinalis* L1-82 (GCF_900537995.1), *Subdoligranulum variabile* DSM 15176 (GCF_000157955.1), *A. baumannii* (GCF_025995075.1), *P. aeruginosa* (GCF_000006765.1), and *S. enterica* (GCF_000439255.1).

### Reconstruction of microbial metabolic network models

Genome-scale models were reconstructed using KBase and CarveMe, and then refined with DEMETER (27, 28, 46). Prior to DEMETER refinement, taxonomic information, Gram status, and MicrobeID were compiled for each microorganism. DEMETER optimization included gap filling, removal of futile cycles, and adjustment of the biomass equation according to Gram status (27). CarveMe and KBase were used with default parameters, and DEMETER was used with its default settings.

### Quality validation of metabolic network models

Stoichiometric consistency and flux consistency were assessed to evaluate the accuracy of the microbial metabolic network models. Stoichiometric consistency is a key indicator for verifying model accuracy, as it ensures that the molecular mass is always positive and mass is conserved on each side of a reaction. Incorrect stoichiometry in reactions can lead to missing metabolic products, thereby affecting the predictive accuracy of the model. Flux consistency was evaluated using the findFluxConsistentSubset function in the COBRA Toolbox, which identifies flux-inconsistent reactions. Analyses were performed in MATLAB R2018a with the COBRA Toolbox v3.0.

Model predictions were evaluated against experimentally curated secretion/uptake data from the NJC19 data set (31). Predictions were matched to NJC19 annotations at the metabolite-exchange level, and accuracy was computed as follows:


$$ACC=\frac{TP+TN}{TP+TN+FP+FN}$$

where TP = true positives, TN = true negatives, FP = false positives, and FN = false negatives. Predictions were obtained using Python 3.9 with COBRApy 0.29.0.

### Identification and screening of exogenous nutrients

The prediction of exogenous nutrients was conducted using the SCC algorithm. An SCC represents the largest set of nodes where every pair of nodes is connected by at least one path. This method constructs a directed acyclic graph, in which nodes represent different components and edges represent connections between components. Initially, all reactions were extracted from GEMs, and based on the relationships between substrates and products within these reactions, the metabolic network was transformed into a bipartite graph. Using the DiGraph function from the Python package NetworkX (v3.2.1, Python 3.9), this bipartite graph was converted into a directed graph. The directed graph was then decomposed into several SCCs using the strongly_connected_components function. In the process of identifying essential nutrients, SCCs with no incoming edges but at least one outgoing edge were first identified and defined as “source components.” All metabolites within these source components were considered potential key nutrients. SCC-identified source components were limited to ≤5 metabolites per set and are hereafter termed candidate exogenous nutrients (38). This threshold, chosen to reflect realistic candidate exogenous-nutrient set composition, excludes on average only 3.3% of candidates; larger caps (e.g., 10 or 100) do not materially change outcomes.

FVA was performed on exchange reactions corresponding to the screened candidate exogenous nutrients. Metabolites whose exchange reactions had a minimum FVA flux <0 were treated as having potential uptake capacity under the tested medium conditions; candidates meeting this criterion were retained as exogenous nutrients for drug derivatization. Further examination was conducted to determine if these selected exogenous nutrients contained functional groups such as amino, carboxyl, or hydroxyl groups. Exogenous nutrients containing at least one of these functional groups were chosen as candidates for experimental modification of exogenous metabolites.

### Assay of growth curves

M9 medium was optimized by adjusting glucose and NH_4_Cl, yielding a composition of 0.1% glucose, 0.678% Na_2_HPO_4_, 0.3% KH_2_PO_4_, 0.1% NaCl, 0.0482% MgSO_4_, 0.1% NH_4_Cl, and 0.0022% CaCl_2_, with pH set to 7.0 and a total volume of 1,000 mL. In preliminary experiments, a concentration gradient (0, 0.1, 0.5, 1, 5, and 10 mM) was tested over 24 h with OD_600_ measurements taken every 4 h. Final growth curve assays used concentrations of 0.1, 0.25, 0.5, 0.75, and 1 mM for acetamide and succinic acid, and 0.1, 0.5, 1, 1.5, and 2 mM for urea, with equimolar DMSO and sterile blank controls included. The experimental procedure was performed as follows.

Under sterile conditions, a single colony was inoculated into 20 mL Luria–Bertani (LB) medium and cultured at 37°C with shaking for 7–8 h until OD_600_ ≈ 0.8; an equal volume of 50% glycerol was then added, and the culture was stored at −20°C. A 0.1% inoculum was subsequently transferred to 5 mL minimal medium and incubated under the same conditions for 6–8 h. Exogenous nutrients at various concentrations (0 mM as control) were prepared in triplicate. To prepare the assay, 3 mL of restrictive medium was mixed with 3 mL of bacterial culture to yield a 1% inoculum; then, 50 μL of each nutrient concentration was added to sterile tubes, followed by 4.95 mL of the inoculum, and 0.1 mL of the final mixture was dispensed into a 96-well plate for growth curve measurement every 4 h.

### Activity inhibition assay

LB medium served as the growth medium. To evaluate the inhibition effects, three replicates and a control group were set up for each inhibitory concentration. DMSO was maintained at 1% of the culture volume (including the control) to minimize its effects. An aseptic group was additionally set up within the experimental groups to ensure no contamination occurred during the culture process. Bacteria were activated on sterile plates, incubated overnight at 37°C, and a single colony was then cultured in a cell culture flask with shaking at 37°C (160 rpm) until OD_600_ reached 0.6–0.8. Growth was monitored by measuring OD_600_ every 12 h over a 72 h period using 2 mL samples in sterile 5 mL centrifuge tubes.

The calculation formula for the inhibition rate is as follows:


$$Inhibition\;rate=\frac{OD_{600,\;control}-OD_{600,\;experimental}}{OD_{600,\;control}}\times 100\%$$

where OD_600, control_ is the optical density at 600 nm measured for the control group and OD_600, experimental_ is the optical density at 600 nm measured for the experimental group.

GraphPad Prism v8 was applied to fit the inhibition rates of the two drugs and calculate their respective IC_50_ values by nonlinear regression (variable slope).

## Data Availability

The GEMs for seven microorganisms, which are used to select modeling methods, and for three pathogenic bacteria, which are used to validate exogenous nutrient strategies in combination with existing antimicrobial agents, are available on GitHub at https://github.com/hzauzqy/Exogenous-Nutrients. Experimental data are provided to evaluate the performance of these GEMs.

## SUPPLEMENTAL MATERIAL

Supplemental Material msystems.01586-25-s0001.docx Supplemental text, figures, and tables.

Supplemental Data msystems.01586-25-s0002.xlsx Flux scores.

ASM does not own the copyrights to Supplemental Material that may be linked to, or accessed through, an article. The authors have granted ASM a non-exclusive, world-wide license to publish the Supplemental Material files. Please contact the corresponding author directly for reuse.
