# Supplementary material for: Screening of exogenous nutrients for pathogenic bacteria and development of highly active bactericides
Source: mSystems. 2026 Feb 12;11(3):e01586-25. doi: 10.1128/msystems.01586-25 (PMC13011427; doi:10.1128/msystems.01586-25)
Supplement: Supplemental Material — Supplemental text, figures, and tables. [file msystems.01586-25-s0001.docx]

**Supplementary** **Methods**

Conjugates were prepared by HATU/DMAP-mediated amide coupling (DMF, 38–50 °C) with DIPEA as base unless otherwise noted; reactions were monitored by TLC/HPLC and purified by silica gel chromatography. Full screens of coupling agents, bases, solvents, and temperatures are provided in Tables S1–S2.

All target compounds were assessed for purity by HPLC (Agilent 1200 Series; Agilent Technologies). Separations were carried out on an Agilent TC-C18(2) column (4.6 × 250 mm, 5 µm) under isocratic conditions. For NC, NA, and MA, the mobile phase was HPLC-grade methanol/ultrapure water (50:50, v/v); for MN, methanol/0.1% trifluoroacetic acid in water (70:30, v/v). HPLC traces for all four compounds are shown in Supplementary Figures S23–S26; purities and retention times (t_R_) were as follows: NC, 97.2% (t_R_ = 5.6 min); NA, 98.7% (t_R_ = 10.2 min); MA, 98.8% (t_R_ = 10.2 min); MN, 98.5% (t_R_ = 15.0 min).

**Synthesis of target compound NA**

The synthesis of target compound NA was conducted as follows: Add N (300.12 mg, 1.29 mmol, 1.0 eq), DMAP (188.6 mg, 1.55 mmol, 1.2 eq), HATU (539.56 mg, 1.55 mmol, 1.2 eq), and NaH (100.5 mg) sequentially to a 100 mL two-neck flask to create an alkaline environment. Then, 5 mL of DMF was added and the mixture was stirred at 38°C under nitrogen protection for 1 hour. AM dissolved in DMF (91.56 mg, 1.55 mmol, 1.2 eq) was injected into the reaction system, and the temperature was raised to 50°C. After 24 hours, the reaction was quenched with 10 mL of ice water. The mixture was extracted three times with ethyl acetate (50 mL each), washed three times with saturated sodium chloride solution (50 mL each), and dried over anhydrous sodium sulfate. The solvent was removed by rotary evaporation, followed by purification using column chromatography (mobile phase of petroleum ether acetate = 1:3). A pale-yellow solid weighing 162.1 mg was obtained as the product NA.

**Synthesis of target compound MA**

The synthesis of intermediate was carried out as follows: Succinic anhydride (300.12 mg, 3.00 mmol) and DMAP (439.81 mg, 3.6 mmol) were sequentially added to a 100 mL two-neck flask, followed by the addition of 10 mL of dichloromethane. The reaction was stirred at 38°C for 1 hour. Subsequently, AM (212.49 mg, 3.6 mmol) dissolved in 3 mL of DMF and 1 mL of N,N-Diisopropylethylamine (DIPEA) were added, and the reaction was continued at 50°C for 36 hours until completion. After concentration under reduced pressure, the residue was dissolved in ethyl acetate, and the solution was extracted three times with ethyl acetate (50 mL × 3) and washed three times with saturated sodium chloride solution (50 mL × 3). The organic layer was dried over anhydrous sodium sulfate, and the solvent was removed under reduced pressure to yield a yellow oily compound. The product was further dried in a vacuum oven at 50°C for 8 hours for later use.

The specific reaction steps for synthesizing the target compound MA are as follows: Intermediate, CDI (197.32 mg, 1.13 mmol), and 5 mL of dichloromethane were sequentially added to a 100 mL two-neck flask. The reaction mixture was stirred at 38°C for 2 hours, followed by the addition of M (300.02 mg, 1.13 mmol) and DIPEA (1 mL). The reaction mixture turned white, and its progress was monitored using TLC with a developing solvent of (V_MeOH_ : V_DCM_ =1 : 60). After 24 hours, the reaction was complete. The mixture was extracted three times with dichloromethane (50 mL × 3) and washed three times with saturated sodium chloride solution (50 mL × 3). The organic layer was dried over anhydrous sodium sulfate, and the solvent was removed under reduced pressure. The crude product was purified by column chromatography using (V_MeOH_:V_DCM_ =1:100) as the mobile phase. After further removal of the solvent by rotary evaporation, the product was dried under vacuum at 40°C for 8 hours to yield 147.2 mg of a pale-yellow oily compound, with a yield of 32.1%.

**Synthesis of target compound MN**

The specific synthesis steps for the target compound MN are as follows: Monoethyl succinate (96.26 µL, 750.92 mmol), N-(3-dimethylaminopropyl)-N'-ethylcarbodiimide hydrochloride (EDC) (159.51 µL, 901.10 mmol), and 4-methylmorpholine (NMM) (101.27 µL, 901.10 mmol) were added to 5 mL of DCM. The mixture was stirred at room temperature for 1 hour until dissolved, after which M (200 mg, 750.92 mmol) was added. The reaction was allowed to proceed at room temperature for 24 hours, followed by quenching with 5 mL of ice-cold brine. The mixture was washed with brine and DCM three times, dried over anhydrous sodium sulfate, and concentrated using a rotary evaporator. The crude product was purified by column chromatography (V_MeOH_:V_DCM_ =1:100) and further dried under vacuum at 50°C for 5 hours, yielding approximately 145 mg of a pale yellow oily product MN, with a yield of 48.95%.

**Synthesis of target compound NC**

The specific reaction steps for synthesizing compound NC are as follows: In a 100 mL two-neck flask, N (300.1 mg, 1.29 mmol), HATU (589.2 mg, 1.55 mmol), and DMAP (189.2 mg, 1.55 mmol) were sequentially added, followed by 5 mL of DCM. The reaction mixture was stirred at 40°C for 1 hour. Subsequently, CA (232.3 mg, 3.87 mmol, 3 eq) dissolved in DMF and DIPEA (1.45 mL, 6.45 mmol, 5 eq) were added to the reaction solution. The temperature was then increased to 50°C, and the reaction was monitored using thin-layer chromatography (TLC) with a developing solvent of V_PE_:V_EA_=1:2. After 48 hours, the reaction was quenched with 10 mL of ice-cold brine. The mixture was washed three times with saturated sodium chloride solution (50 mL × 3) and extracted 3-5 times with ethyl acetate (50 mL × 3). The organic layer was dried over anhydrous sodium sulfate, concentrated using a rotary evaporator, and purified by column chromatography (V_PE_:V_EA_=1:2). The final product was dried under vacuum at 50°C for 5 hours, yielding 91.3 mg of a pale yellow solid, corresponding to a yield of 20.9%.

**Supplementary** **Figures**


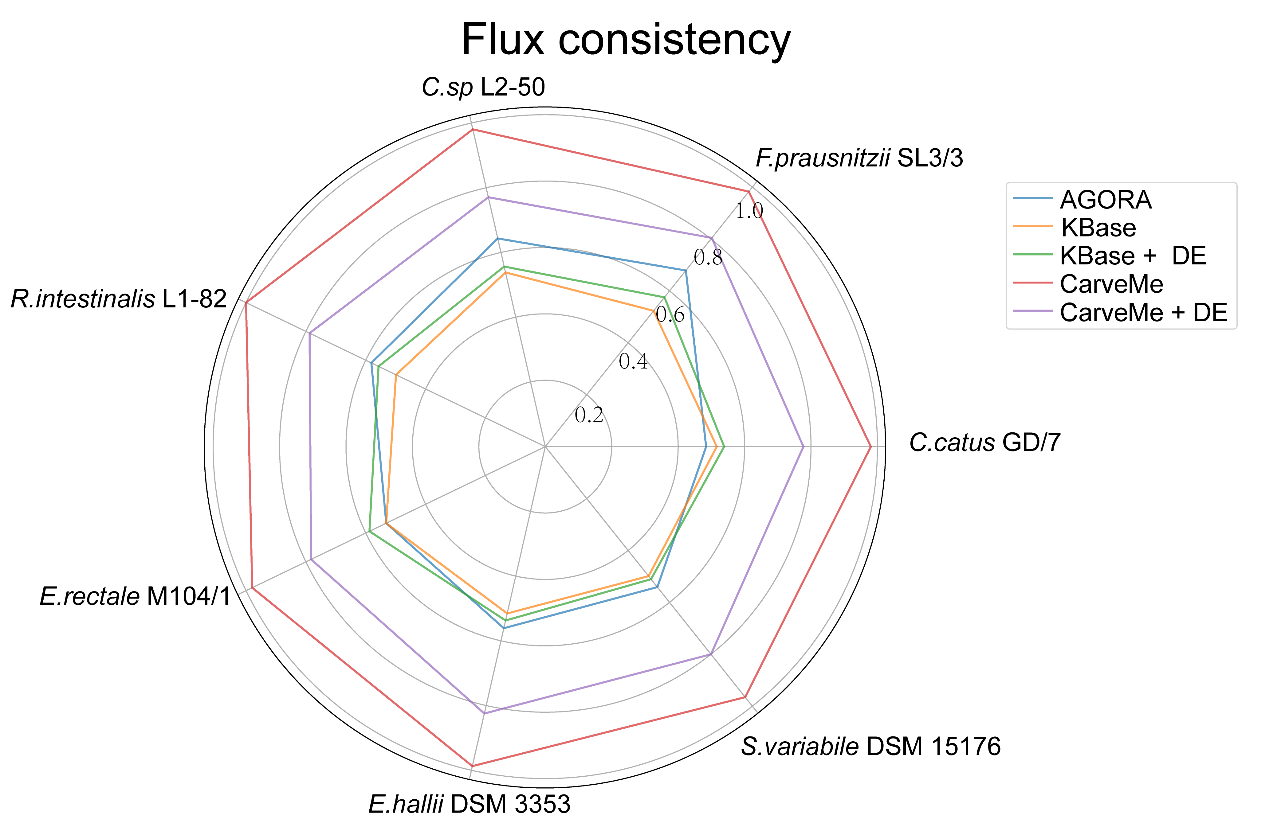


**Fig. S1. Consistency analysis of seven bacterial GEM flux consistency using different modeling methods.**


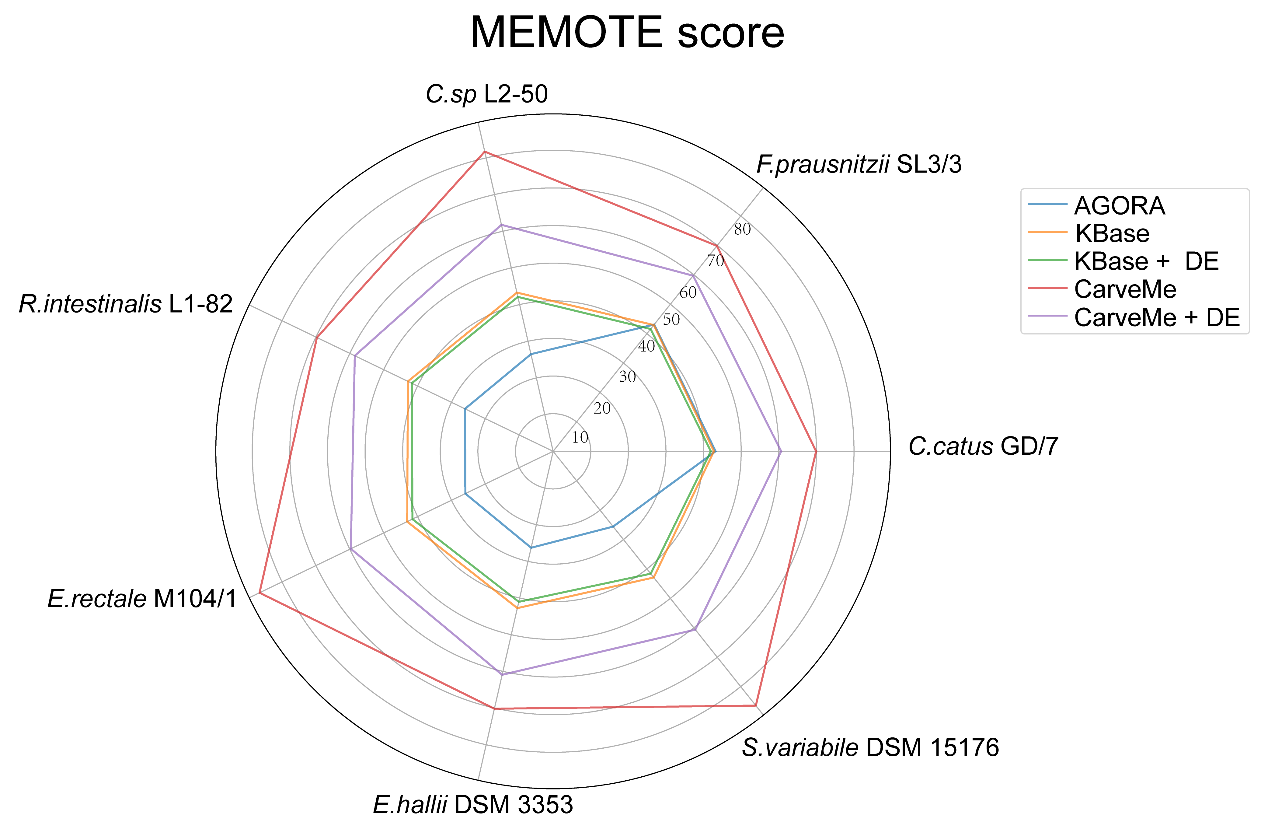


**Fig. S2. Consistency analysis of seven bacterial GEM MEMOTE score using different modeling methods.**


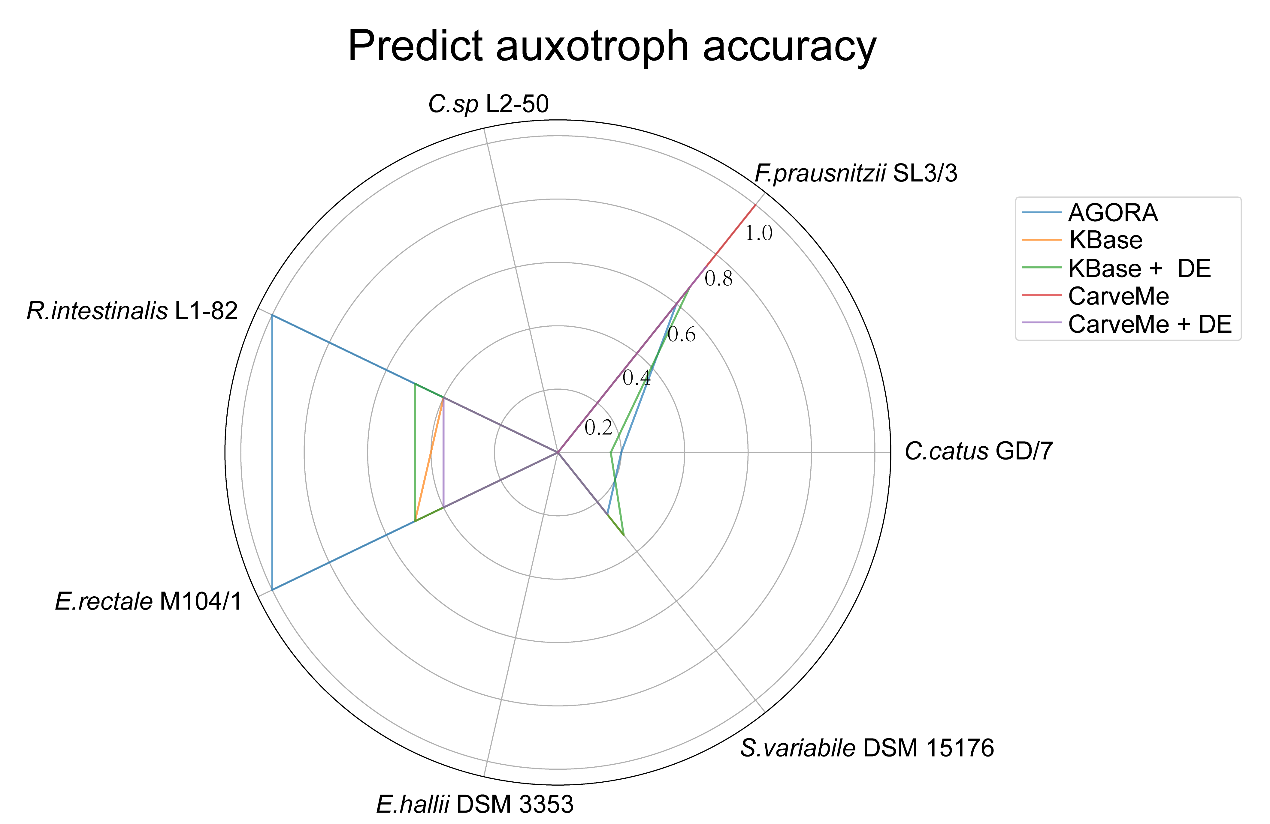


**Fig. S3. Consistency analysis of seven bacterial GEM predict auxotroph accuracy score using different modeling methods.**


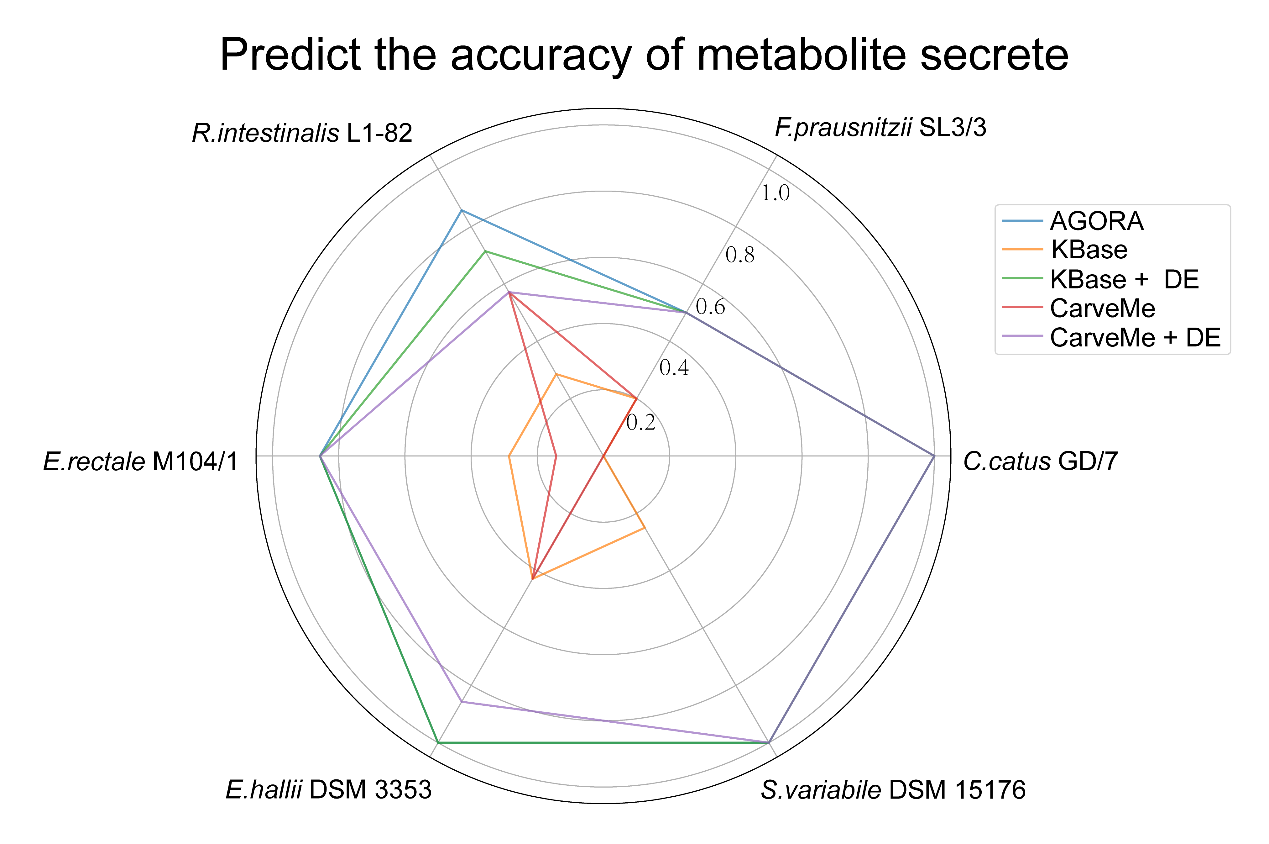


**Fig. S4. Consistency analysis of seven bacterial GEM predict the accuracy of metabolite secrete using different modeling methods.**


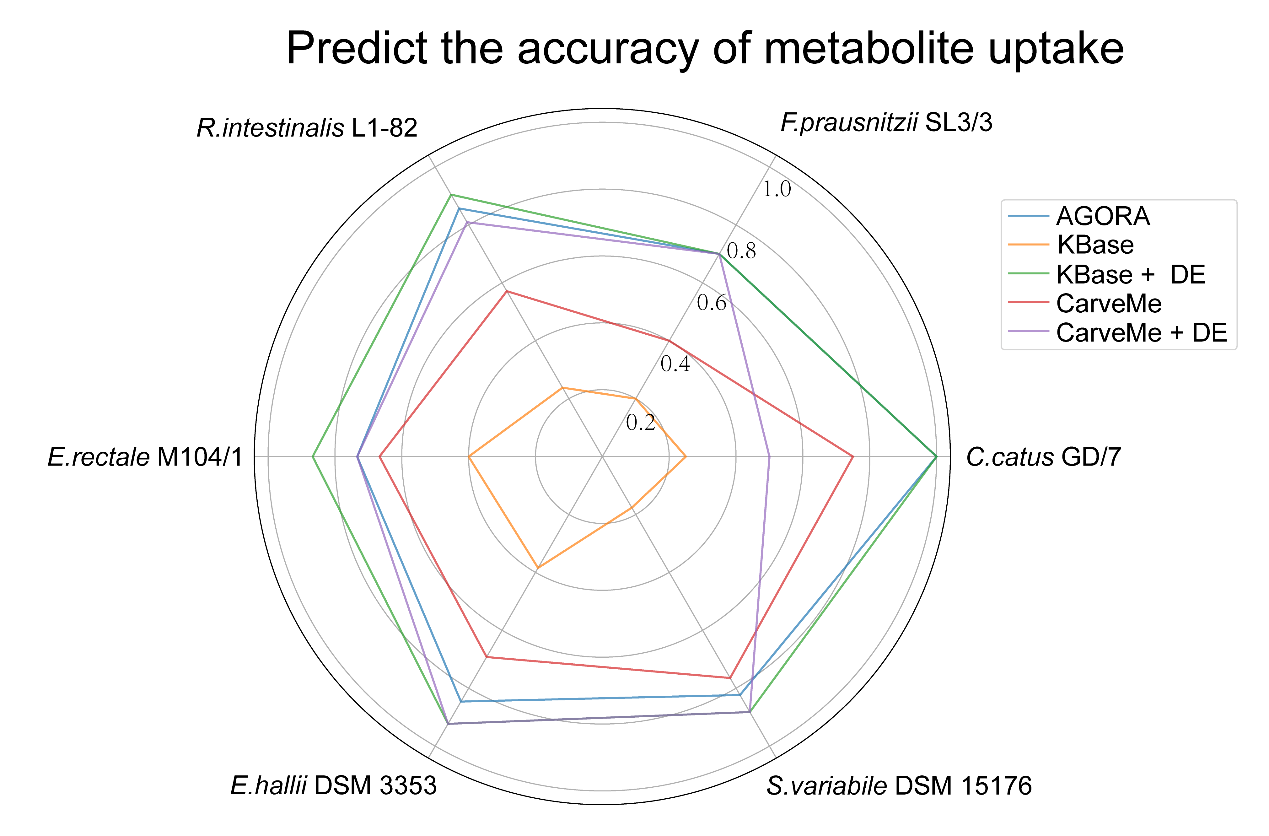


**Fig. S5. Consistency analysis of seven bacterial GEM predict the accuracy of metabolite uptake using different modeling methods.**


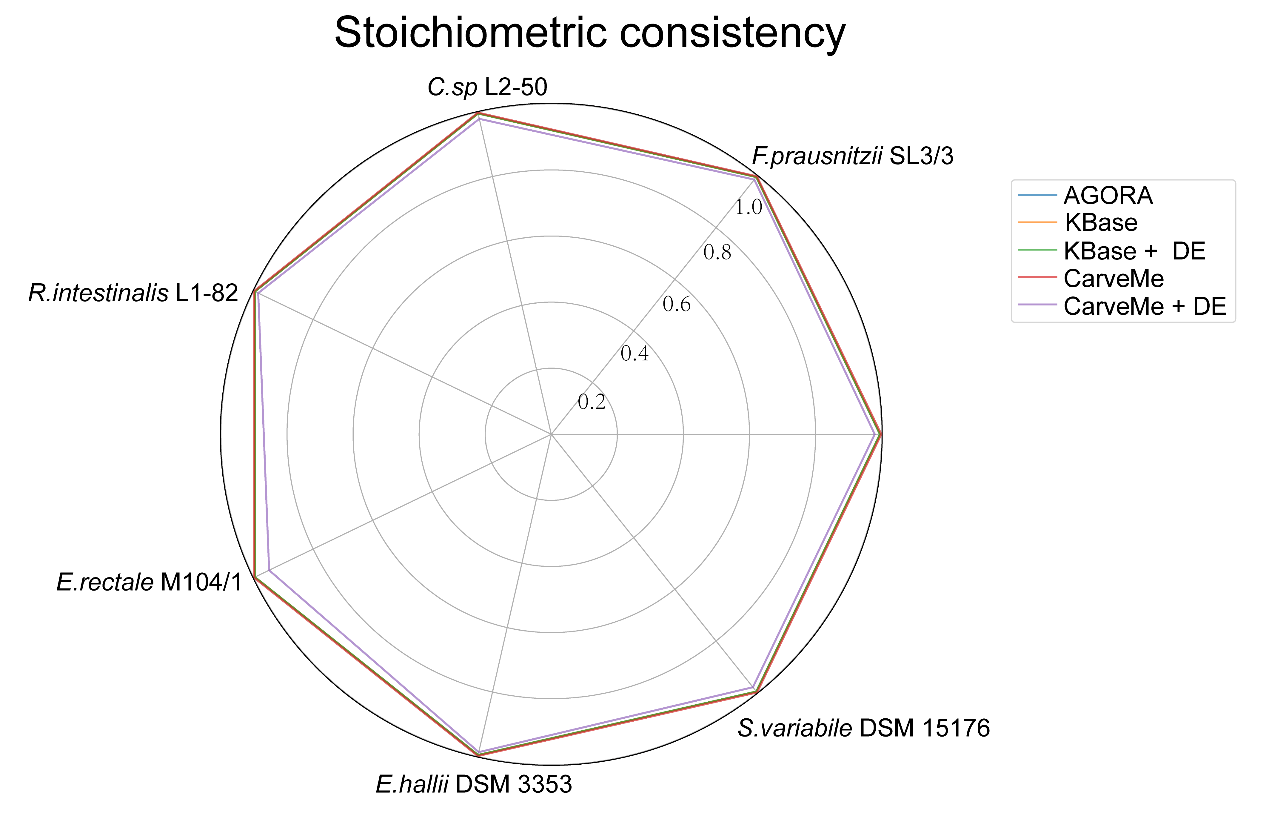


**Fig. S6. Consistency analysis of seven bacterial GEM stoichiometric consistency using different modeling methods.**


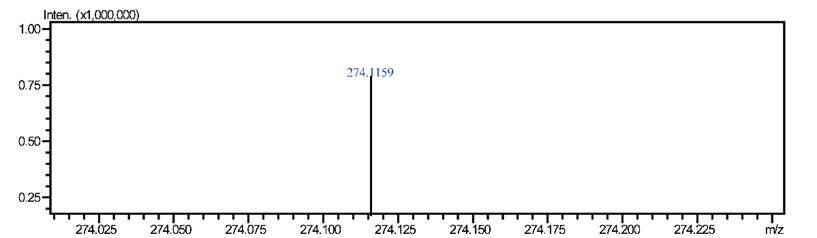


**Fig. S7. The mass spectrum of compound NA.**


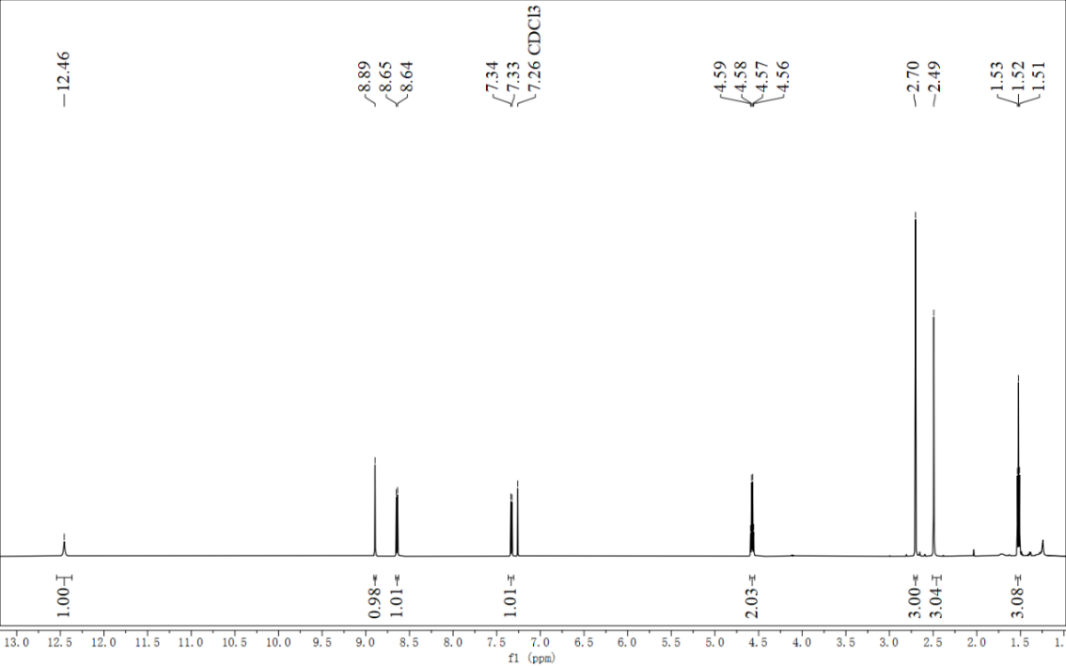


^1^H NMR (600 MHz, CDCl_3_) δ 12.39 (s, 1H), 8.83 (s, 1H), 8.58 (d, *J* = 8.1 Hz, 1H), 7.27 (d, *J* = 8.2 Hz, 1H), 4.51 (q, *J* = 7.2 Hz, 2H), 2.64 (s, 3H), 2.43 (s, 3H), 1.46 (t, *J* = 7.2 Hz, 3H).

**Fig. S8. The ^1^H NMR spectrum of compound NA.**


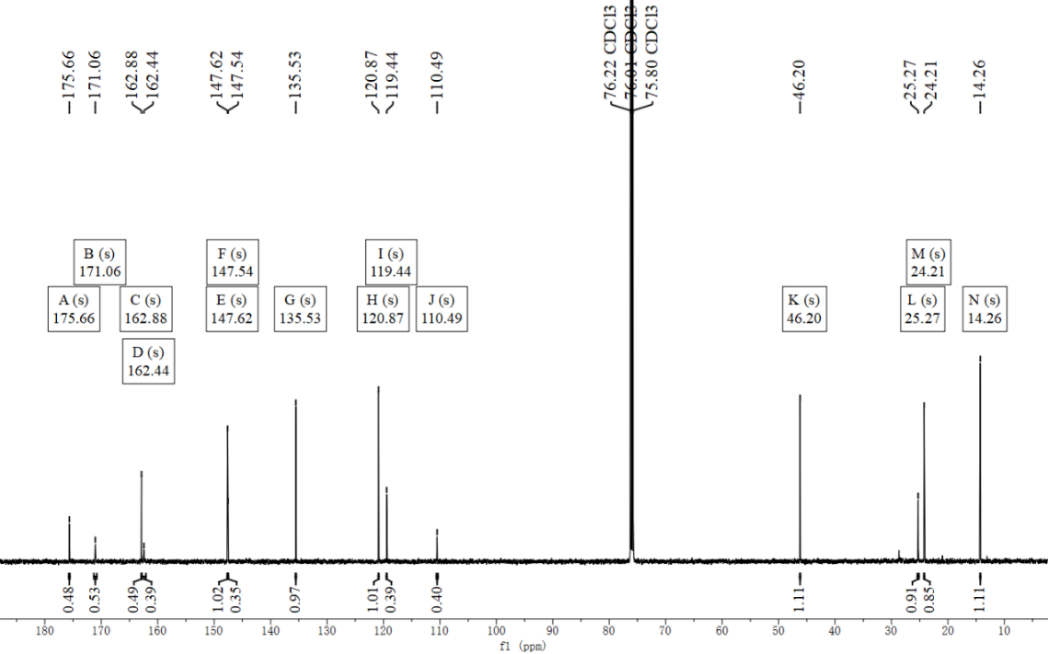


^13^C NMR (150 MHz, CDCl_3_) δ 177.71, 165.67, 163.80, 147.72, 147.24, 135.11, 121.31, 118.18, 108.71, 46.49, 44.72, 24.38, 10.22, 7.60.

**Fig. S9. The ^13^C NMR of compound NA.**


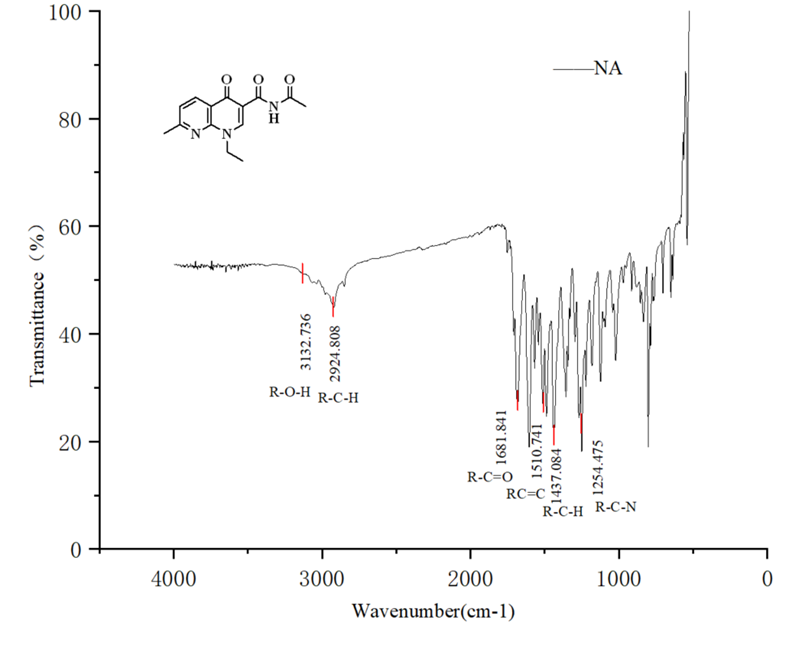


**Fig. S10. The IR of compound NA**


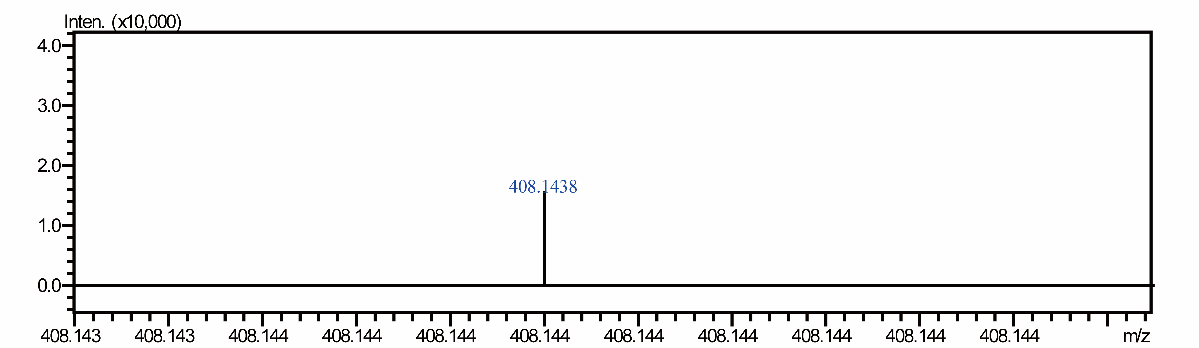


**Fig. S11. The mass spectrum of compound MA.**


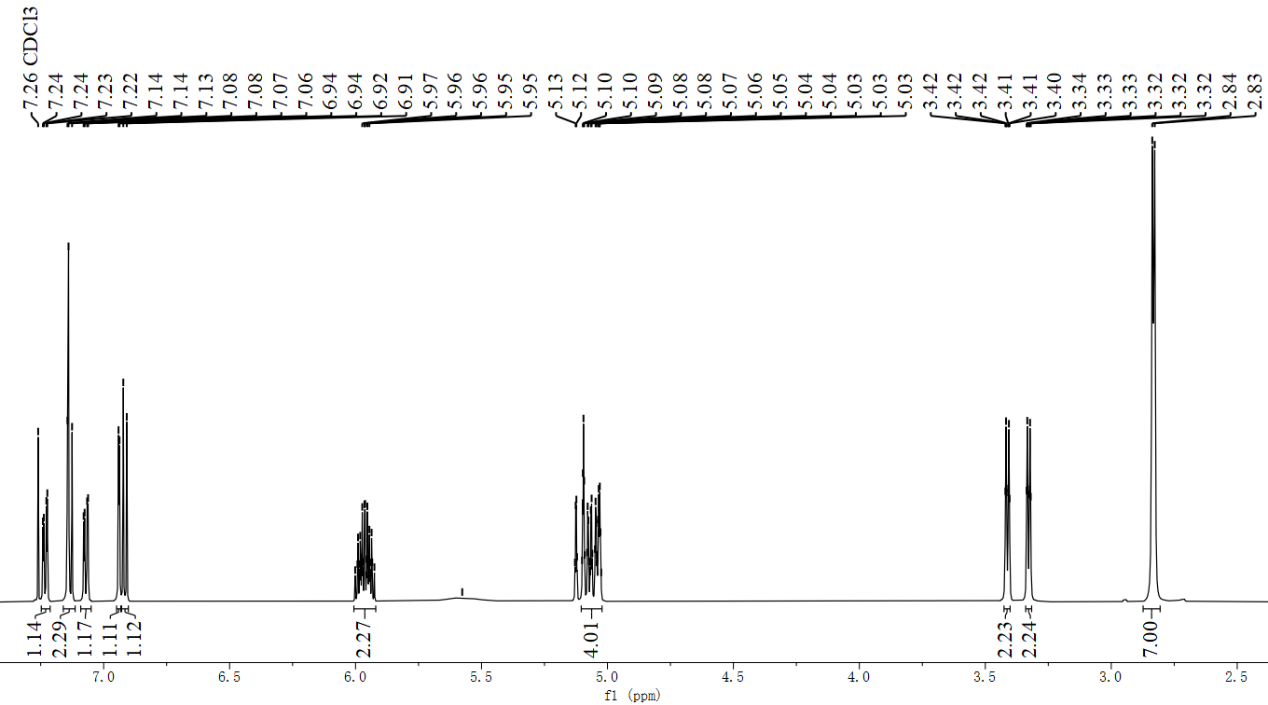


^1^H NMR (600 MHz, CDCl_3_) δ 7.23 (dd, *J* = 8.3, 2.3 Hz, 1H), 7.16 – 7.11 (m, 2H), 7.07 (dd, *J* = 8.3, 2.3 Hz, 1H), 6.94 (d, *J* = 2.3 Hz, 1H), 6.92 (d, *J* = 8.3 Hz, 1H), 5.96 (td, *J* = 16.8, 10.1, 6.7, 5.3 Hz, 2H), 5.10 – 5.02 (m, 4H), 3.43 – 3.40 (m, 2H), 3.33 (dt, *J* = 6.7, 1.6 Hz, 2H), 2.83 (d, *J* = 5.9 Hz, 7H).

**Fig. S12. The ^1^H NMR spectrum of compound MA.**


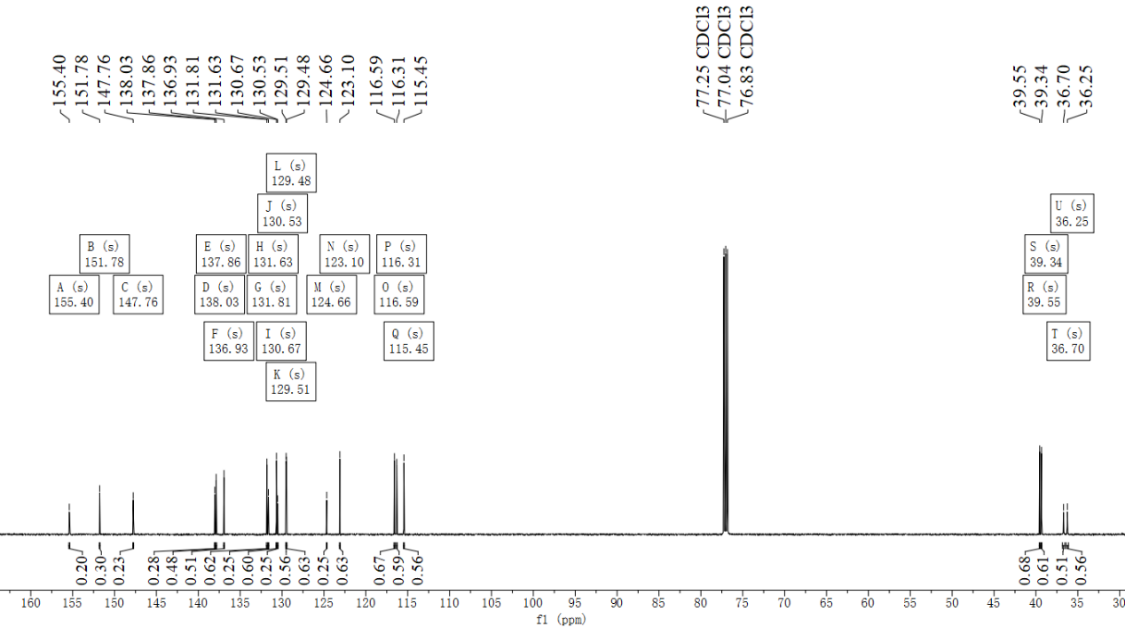


^13^C NMR (150 MHz, CDCl_3_) δ 155.40, 151.78, 147.76, 138.03, 137.86, 136.93, 131.81, 131.63, 130.67, 130.53, 129.51, 129.48, 124.66, 123.10, 116.59, 116.31, 115.45, 39.55, 39.34, 36.70, 36.25.

**Fig. S13. The ^13^C NMR of compound MA.**


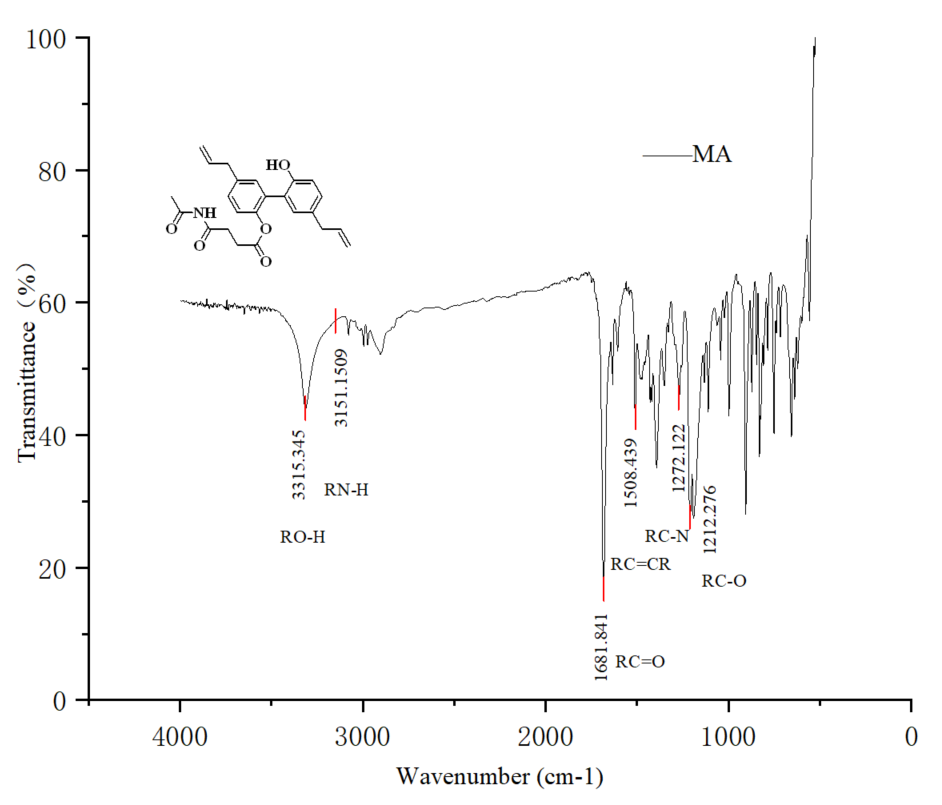


**Fig. S14. The IR of compound MA**


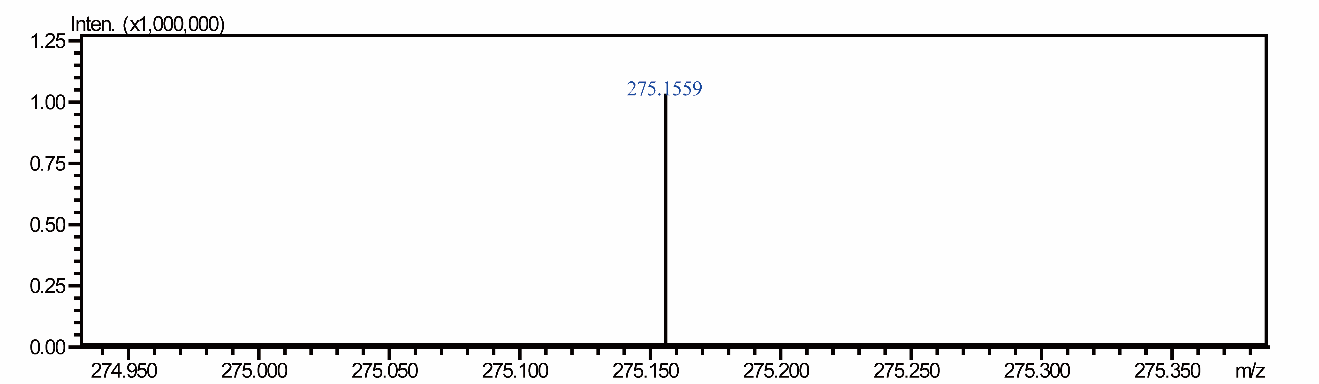


**Fig. S15. The mass spectrum of compound NC.**


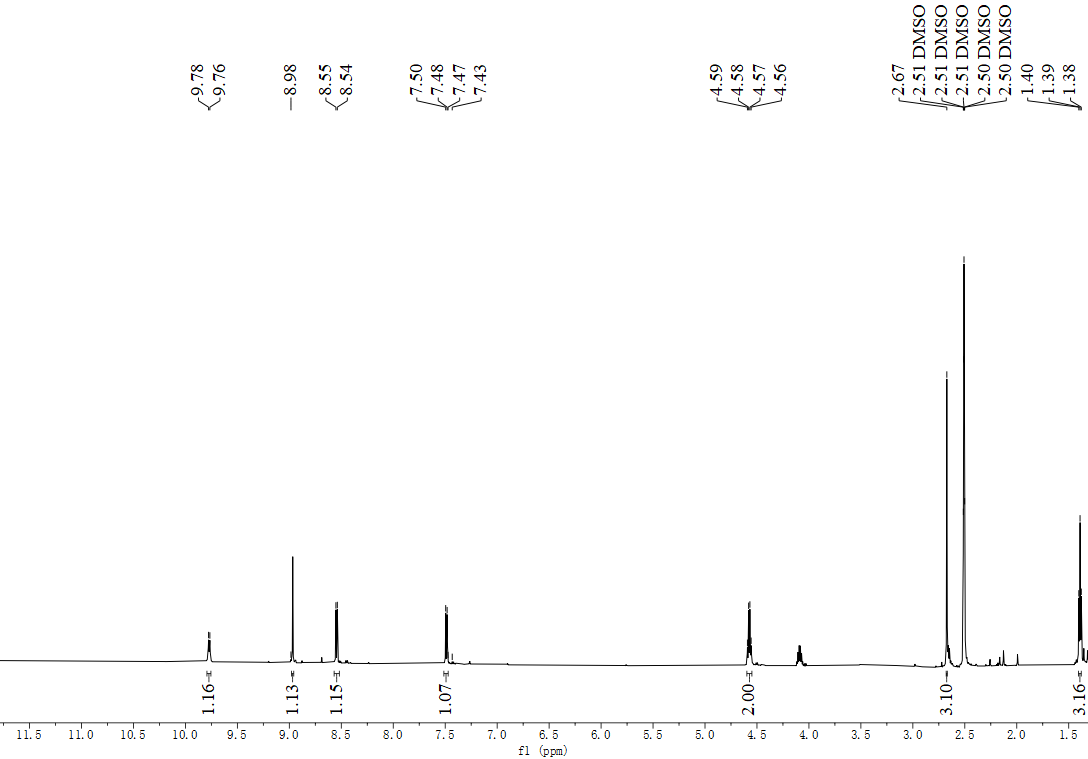


^1^H NMR (600 MHz, DMSO-*d*_6_) δ 9.77 (d, *J* = 7.5 Hz, 1H), 8.97 (s, 1H), 8.54 (d, *J* = 8.1 Hz, 1H), 7.49 (d, *J* = 8.1 Hz, 1H), 4.57 (q, *J* = 7.1 Hz, 2H), 2.67 (s, 3H), 1.40 (d, *J* = 7.1 Hz, 3H).

**Fig. S16. The ^1^H NMR spectrum of compound NC.**


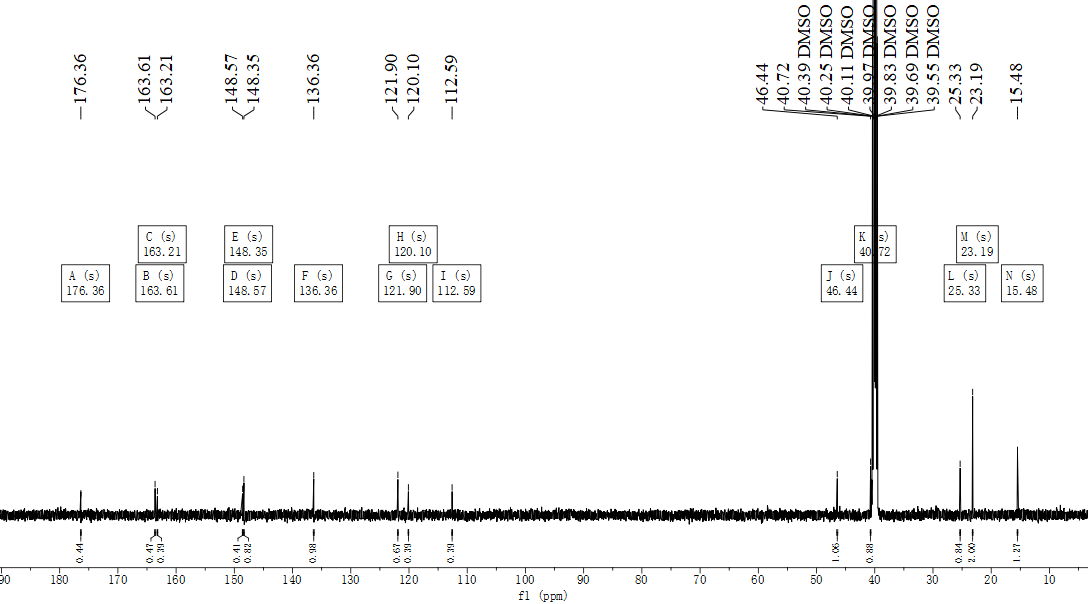


^13^C NMR (150 MHz, DMSO-*d*_6_) δ 176.36, 163.61, 163.21, 148.57, 148.35, 136.36, 121.90, 120.10, 112.59, 46.44, 25.33, 23.19, 15.48.

**Fig. S17. The ^13^C NMR of compound NC.**


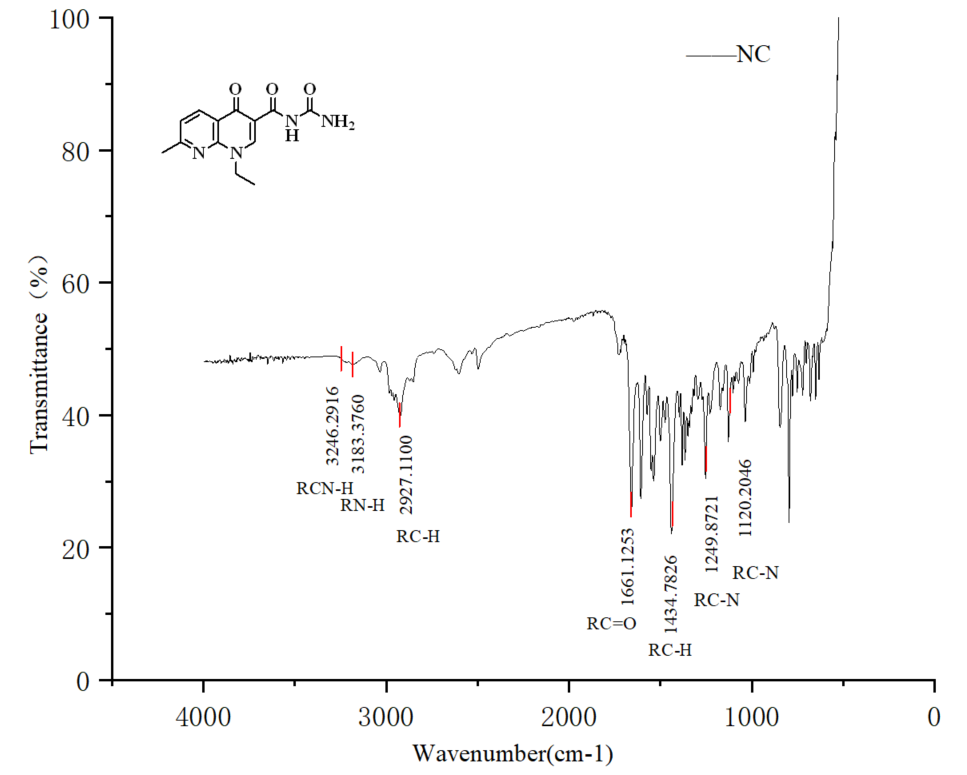


**Fig. S18. The IR of compound NC**

**Fig. S19. The mass spectrum of compound MN.**

**
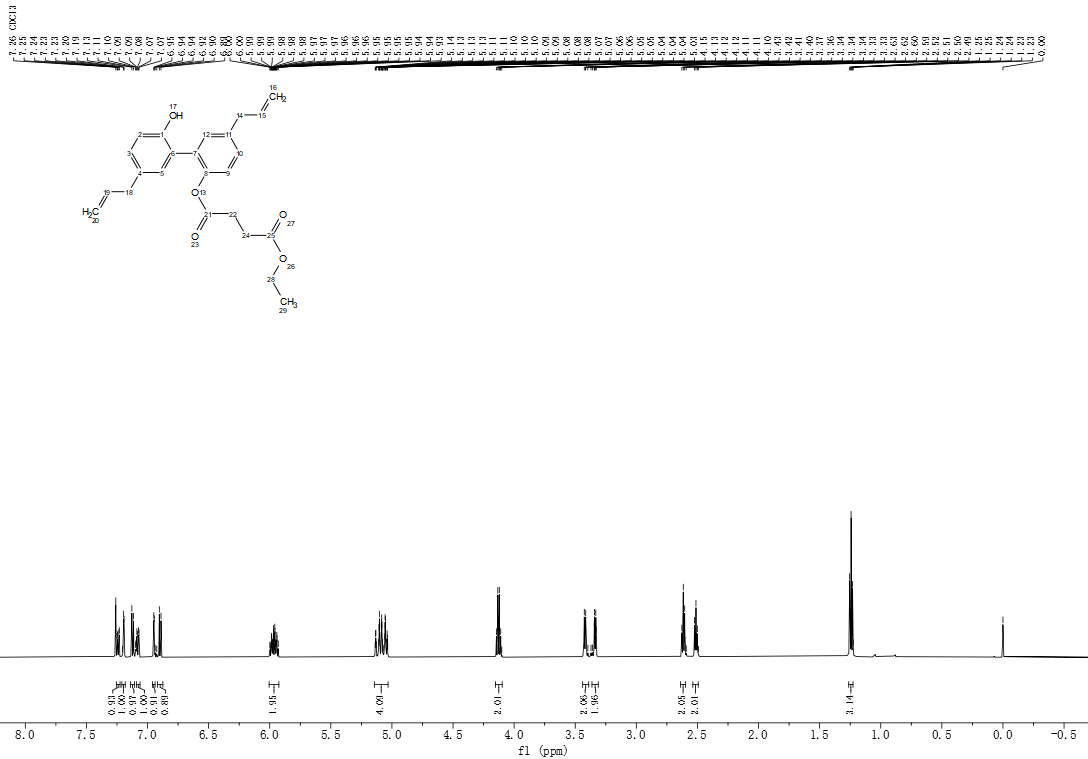
**

^1^H NMR (600 MHz, Chloroform-*d*) δ 7.24 (dd, J = 8.3, 2.2 Hz, 1H), 7.19 (d, J = 2.2 Hz, 1H), 7.12 (d, J = 8.3 Hz, 1H), 7.08 (dd, J = 8.3, 2.4 Hz, 1H), 6.95 (d, J = 2.2 Hz, 1H), 6.90 (d, J = 8.2 Hz, 1H), 6.01 – 5.93 (m, 2H), 5.14 – 5.03 (m, 4H), 4.13 (q, J = 7.1 Hz, 2H), 3.44 – 3.39 (m, 2H), 3.36 – 3.31 (m, 2H), 2.62 (t, J = 6.9 Hz, 2H), 2.51 (t, J = 6.7 Hz, 2H), 1.24 (t, J = 7.1 Hz, 3H).

**Fig. S20. The ^1^H NMR spectrum of compound MN.**


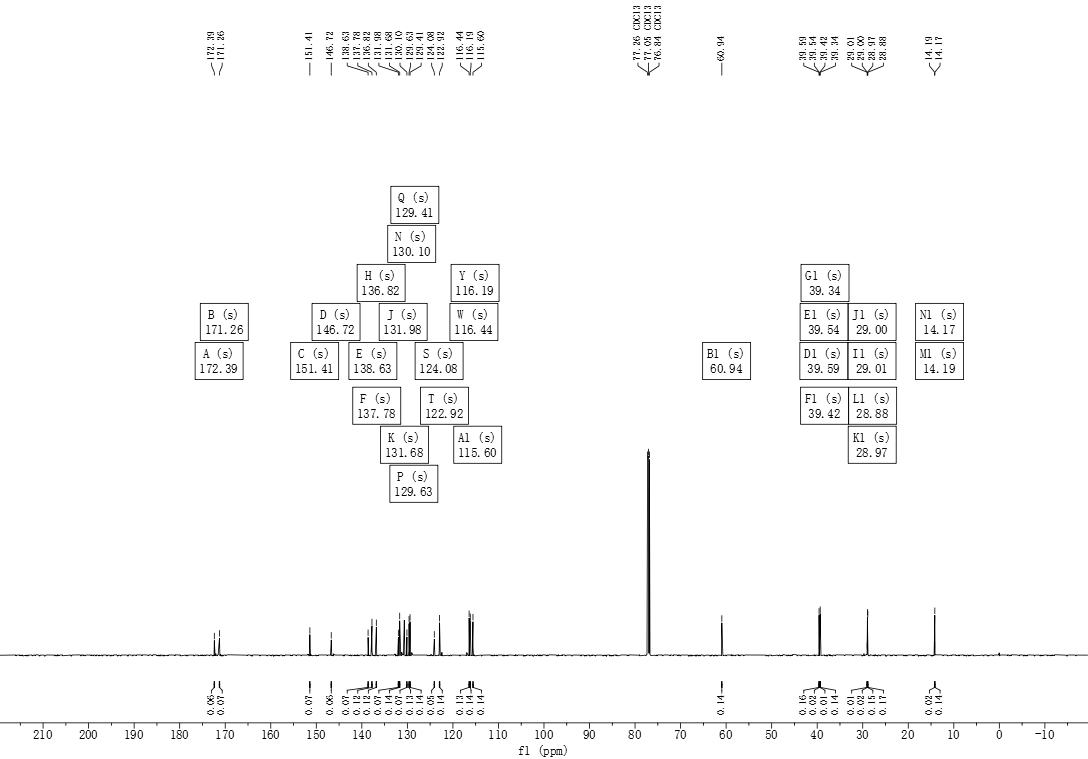


^13^C NMR (150 MHz, Chloroform-*d*) δ 172.39, 171.26, 151.41, 146.72, 138.63, 137.78, 136.82, 131.98, 131.68, 130.68, 130.10, 129.63, 129.41, 124.08, 122.92, 116.44, 116.19, 115.60, 60.94, 60.73, 39.59, 39.54, 39.42, 39.34, 28.97, 28.88, 14.19, 14.17.

**Fig. S21. The ^13^C NMR of compound MN.**





**Fig. S22. The IR of compound MN**

**
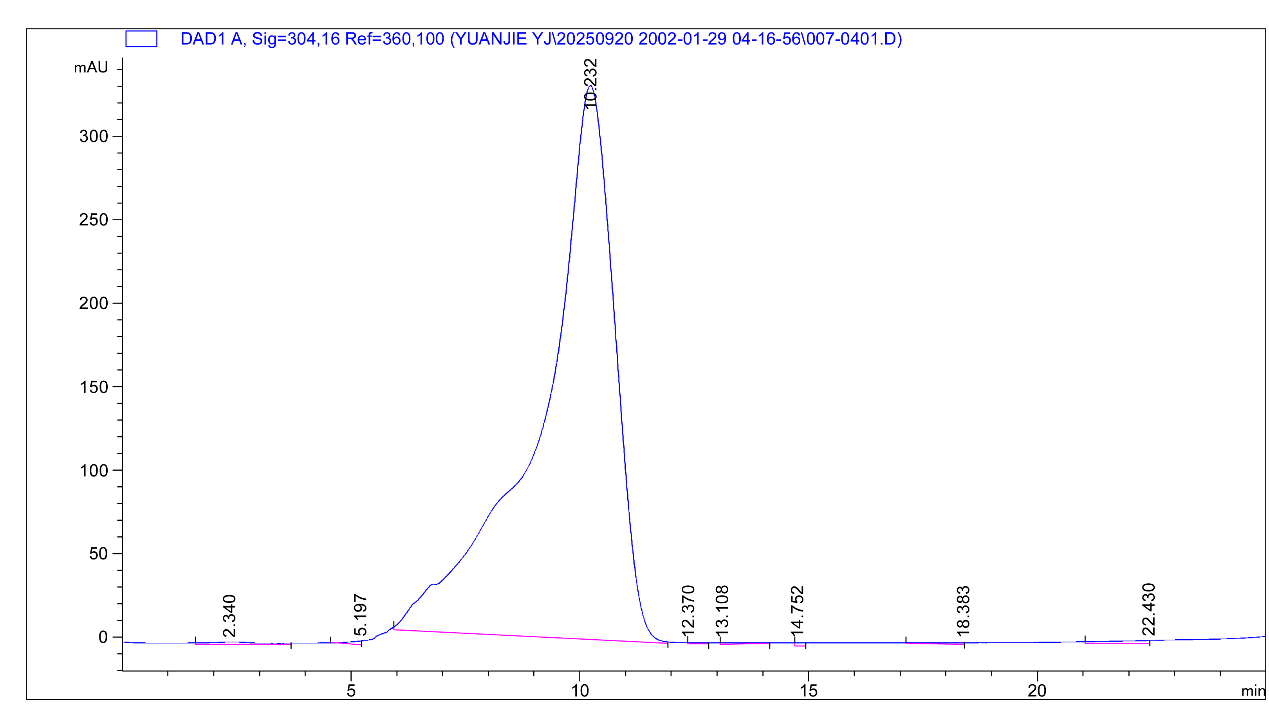
**

**Fig. S23. HPLC chromatogram of MA (purity 98.8%; t_R_ = 10.2 min)**

**
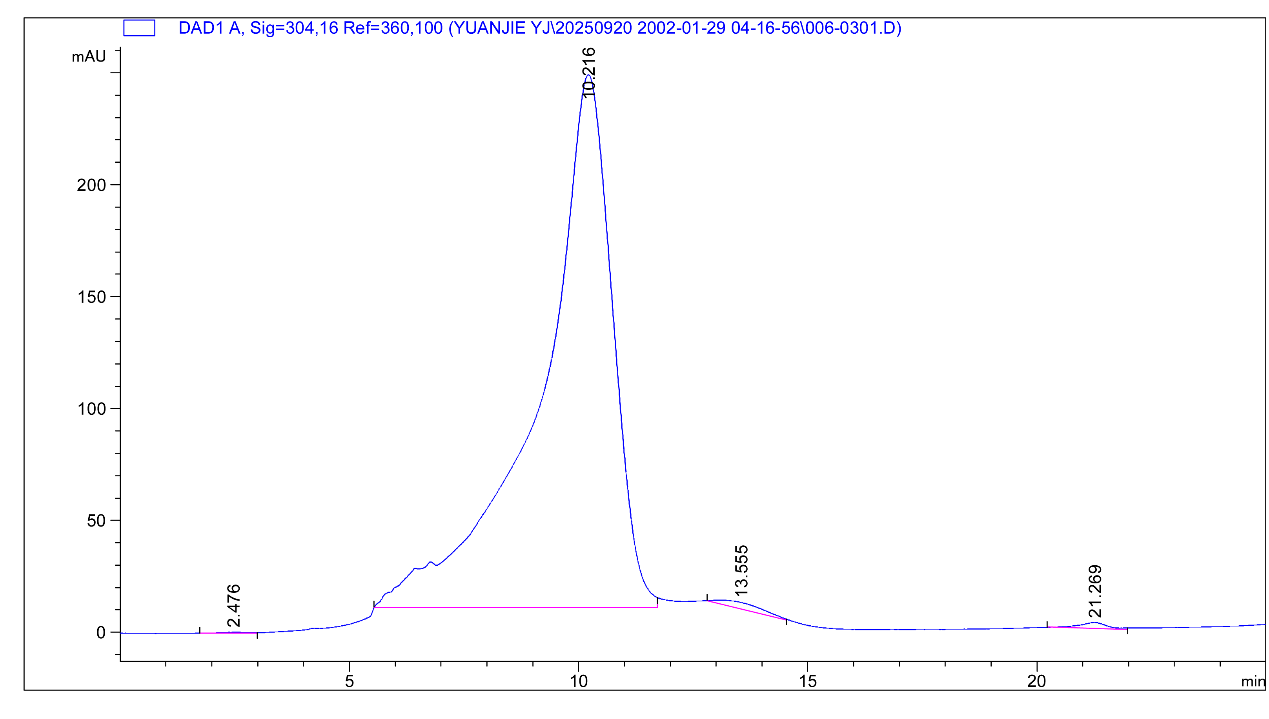
**

**Fig. S24. HPLC chromatogram of NA (purity 98.7%; t_R_ = 10.2 min)**

**
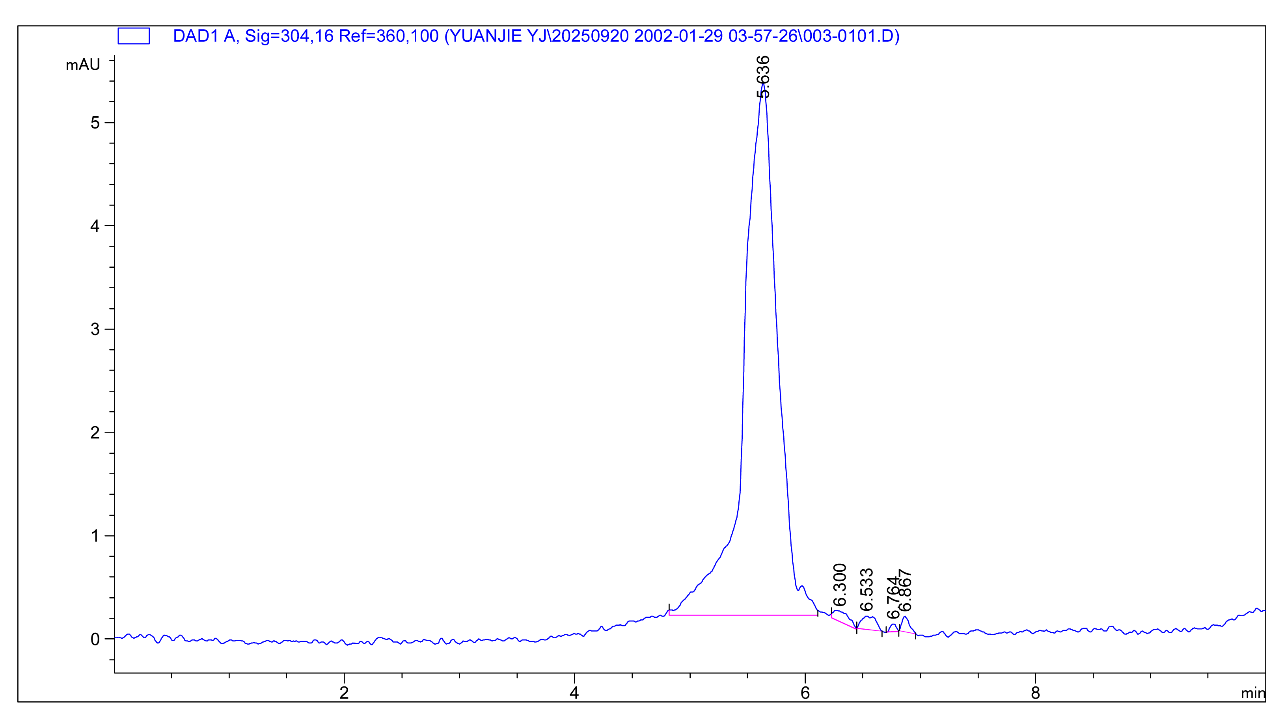
**

**Fig. S25. HPLC chromatogram of NC (purity 97.2%; t_R_ = 5.6 min)**

**
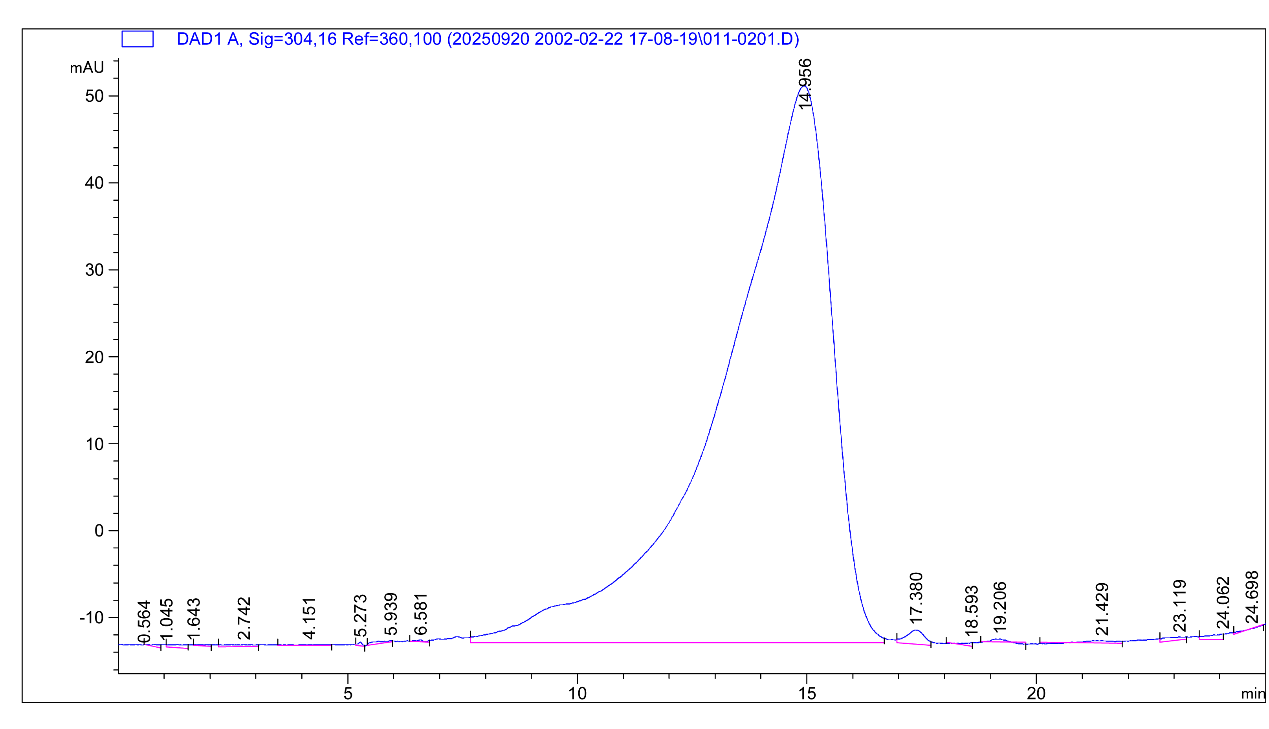
**

**Fig. S26. HPLC chromatogram of MN (purity 98.5%; t_R_ = 15.0 min)**

Tables S1 Screening of coupling agents for NC preparation

| Entry | Coupling Reagent (1.2 equiv) | Solvent | Temperature (°C) | Yield |
| --- | --- | --- | --- | --- |
| 1 | DCC, DMAP | DCM | 38 | N, D |
| 2 | EDCI, DMAP | DCM | 38 | N, D |
| 3 | HATU, DMAP | DCM | 38 | trace |
| 4 | HATU, DMAP | DMF | 50 | trace |

Notes: N.D. = no detectable product under the tested condition; trace = trace formation observed.

Tables S2. Screening of reaction conditions for NA

| Entry | Coupling Reagent (1.1 equiv) | Base | Solvent | Temperature (°C) | Yield |
| --- | --- | --- | --- | --- | --- |
| 1 | HATU, DMAP | DIPEA | DCM | 38 | N, D |
| 2 | HATU, DMAP | NaH | THF | 45 | N, D |
| 3 | HATU, DMAP | NaH | DMF | 50 | Trace |
| 4 | POCl_3_ | PY | DMF | RT | Low |

Notes: N.D. = no detectable product under the tested condition; trace = trace formation observed; low = low isolated yield.
